# Orthogonal functionalization of alternating polyesters: selective patterning of (AB)_*n*_ sequences†
†Electronic supplementary information (ESI) available. See DOI: 10.1039/c9sc03756j


**DOI:** 10.1039/c9sc03756j

**Published:** 2019-09-20

**Authors:** Ni Yi, Thomas T. D. Chen, Junjuda Unruangsri, Yunqing Zhu, Charlotte K. Williams

**Affiliations:** a Chemistry Research Laboratory , Department of Chemistry , University of Oxford , 12 Mansfield Road , Oxford , OX1 3TA , UK . Email: charlotte.Williams@chem.ox.ac.uk; b Department of Chemistry , Imperial College London , South Kensington Campus , London , SW7 1AZ , UK

## Abstract

Precision functionalized polyesters, with defined monomer sequences, are prepared using an orthogonal post-polymerization strategy. These polyesters can be synthesized from bio-derived monomers and are targeted to degrade, by hydrolysis processes, to biocompatible diols and diacids; the new structures enabled by this methodology would be very difficult to synthesize by alternative strategies. A series of 9 well-defined highly alternating AB-type copolyesters, containing terminal and internal alkene functionalities, are synthesized in high conversions by the ring-opening copolymerization of epoxides and cyclic anhydrides. Firstly, the polyesters are functionalized by a selective hydroboration–oxidation reaction to exclusively and quantitatively hydroxylate the terminal alkenes, leaving the alternating internal alkenes unreacted. Subsequently, the internal alkenes are quantitatively transformed into carboxylic acid, amine, alkyl and oligo-ether groups, by thiol–ene reactions, to afford AB polyesters with alternating functional substituents. Three polyesters showing alternating hydrophilic/hydrophobic side-chain sequences self-assemble in solution to form nanostructures that are characterized using transmission electron microscopy and dynamic light scattering methods (*R*_h_ = 100–300 nm). The selective patterning methodology provides facile, efficient and orthogonal functionalization of alternating polyesters with near-quantitative (AB)_*n*_ repeat sequences. The method is expected to be generalizable to other polymers and provides access to completely new AB alternating structures with the potential to exploit ligand multi-valency and adjacency to enhance properties.

## Abstract 2

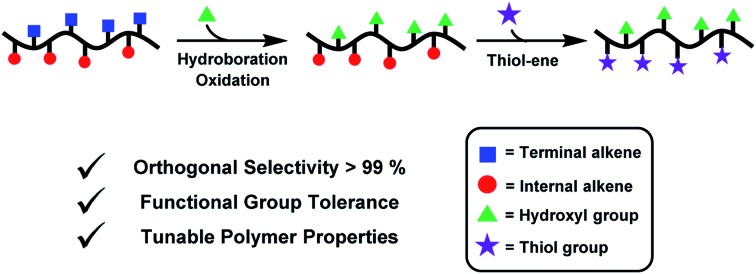
A series of AB alternating polyesters are orthogonally patterned to install two different functionalities at regular intervals along the backbone and with high precision.

## Introduction

Precise control of monomer sequence and accurate placement of functional side-chain substituents are currently key challenges for next-generation polymers.^1^,^2^ Functional precision polymers should show unprecedented spatial organization, folding and self-assembly.^1^,^2^ Applications include targeted cell transfection, tissue engineering, antimicrobials, polymers for information storage and coding.^1^–^4^ Access to these materials should also improve understanding and control over biological performance, structural organization, degradation kinetics and microstructure-macroscopic thermal/mechanical property relationships.^1^–^4^ In this work, a new and generally applicable route to pattern AB alternating polyesters with functional, and orthogonally reactive, side-chain substituents is described. These polyesters are deliberately selected to be hydrolyzed to biocompatible diols and diacids and to utilize monomers that are, or in future could be, sourced from renewables.^5^–^8^ Functionalized, precision AB polyesters are currently very rare,^9^–^12^ and generally such polymers are more challenging to prepare. Sampson and co-workers have pioneered a series of alternating cationic/lipophilic poly(cyclobutylenes) that show higher antimicrobial activity and lower toxicity than random copolymers.^3^,^13^,^14^ Weck and co-workers demonstrated that donor–acceptor AB alternating polymers showed enhanced structural order compared to random copolymers.^15^,^16^ Meyer and co-workers utilized iterative syntheses to provide the first evidence that precision control over lactide-glycolide sequences allow fine control over (bio)degradation and ester hydrolysis rates.^17^–^19^

One reason for the paucity of prior studies of well-defined AB alternating polymers is the need for careful optimization of reactivity ratios or for the selection of specific donor–acceptor monomers so as to bias for alternation.^16^,^20^ In contrast, the ring opening copolymerization (ROCOP) of most epoxides and anhydrides routinely yields highly alternating AB polyesters. Conversions are generally high and the favorable polymerization thermodynamics enable high yields even when substituted epoxides/anhydrides are applied.^21^–^23^ The selection of the catalyst is important to ensure the highly alternating structure and there are now quite a range of catalysts which effectively prevent epoxide homopolymerization.^21^,^24^–^30^ In addition to its impressive sequence selectivity, ROCOP is also amenable to manufacturing at scale since many epoxides and anhydrides are already produced and used by the chemical industry.^22^ One draw-back of ROCOP is that several useful functional groups are incompatible and, in particular, substituents such as hydroxyl, primary/secondary amine, carboxylic acid and other protic species function as chain transfer agents.^22^,^23^ These chemistries can be successfully installed by post-functionalization reactions, perhaps most commonly by the thiol–ene reaction.^28^–^38^ Relevant to this are several reports of its application using aliphatic polycarbonates prepared by CO_2_/epoxide ROCOP.^38^–^50^ For example, Darensbourg and co-workers used it to make amphiphilic block polycarbonates that self-assembled into micelles.^40^,^44^ Our group and that of Koning have exploited it to transform bio-derived alkene-functionalized polycarbonates and multi-functional thiols into scratch resistant coatings.^31^,^38^ So far, these post-functionalization reactions have been used to install a single functional group either onto a polymer or block polymer backbone.^28^–^50^ Last year, Coates and co-workers reported an example of orthogonal functionalization, reacting an AB polyester using both a thiol–ene and a Schiff base reaction (onto an aldehyde functionalized monomer).^12^ Our goal is to develop other generally applicable orthogonal post-functionalization which exploit the naturally high AB enchainment conferred by ROCOP and to prepare polyesters featuring alternating and mutually reactive side-chain substituents.

**Scheme 1:**
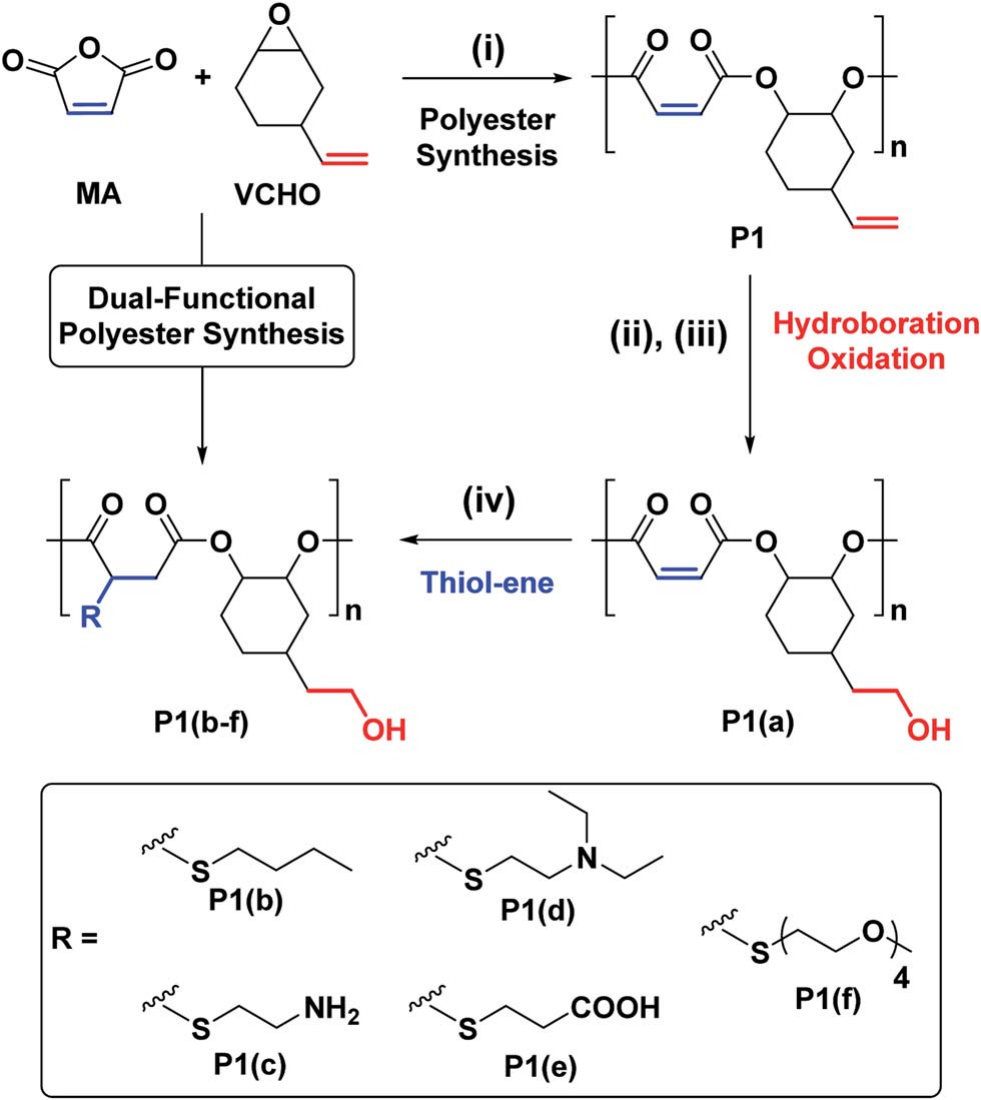
Selective synthesis of dual-functional alternating polyesters (using a combination of MA and VCHO as a representative example) (see ESI† for complete experimental protocols). Reagents and conditions: (i): [SalcyCrCl]/PPNCl/1,2-cyclohexane diol (CHD)/anhydride/epoxide = 1/1/10/100/120, 60 °C, [anhydride]_0_ = 5 M in toluene. (ii): [alkene]_0_/[9-BBN] = 1/1.5, 9-BBN, 1.5 h, 25 °C, [alkene]_0_ = 0.03 M in THF. (iii): [alkene]_0_/[mCPBA] = 1/5.1, mCPBA, 2 h; 25 °C. (iv): [DMPA]/[alkene]_0_/[thiol] = 1/2.5/10, DMPA, thiol, DMSO, UV irradiation at 365 nm (10 W), 2 h, [alkene]_0_ = 0.25 M.

We targeted epoxides and anhydrides featuring alkene substituents as these are widely commercially available and there are already a plethora of efficient alkene transformations. Our attention was drawn to alkene hydroboration–oxidation as an efficient means to install hydroxyl substituents because it has been shown to be compatible with ester linkages, as demonstrated by its successful application to functionalize polyhydroxyalkanoates.^51^–^55^ In the 1980s Brown and co-workers established that primary and secondary alkenes showed up to three orders of magnitude difference in rates of hydroboration when using sterically hindered boranes.^56^,^57^ We reasoned that a polyester containing both primary and secondary alkene groups might undergo selective reaction at the primary alkene sites (Scheme 1). To test this hypothesis, a series of 9 alternating polyesters (**P1–P9**) were synthesized from commercially available alkene functionalized epoxides and anhydrides. The epoxides all feature primary alkene groups, as vinyl-cyclohexene oxide (v-CHO), vinyl-propylene oxide (v-PO) or allyl glycidyl ether (AGE), whilst all the anhydrides feature secondary alkenes either in maleic anhydride (MA), tetrahydro phthalic anhydride (THPA) or a tricyclic anhydride (TCA). Henceforth, the alternating polyester prepared from v-CHO and MA is described as a representative example of the general procedure and characterization methods which are applied to the entire series of new polyesters (Scheme 1(i)).

## Results and discussion

**Scheme 2:**
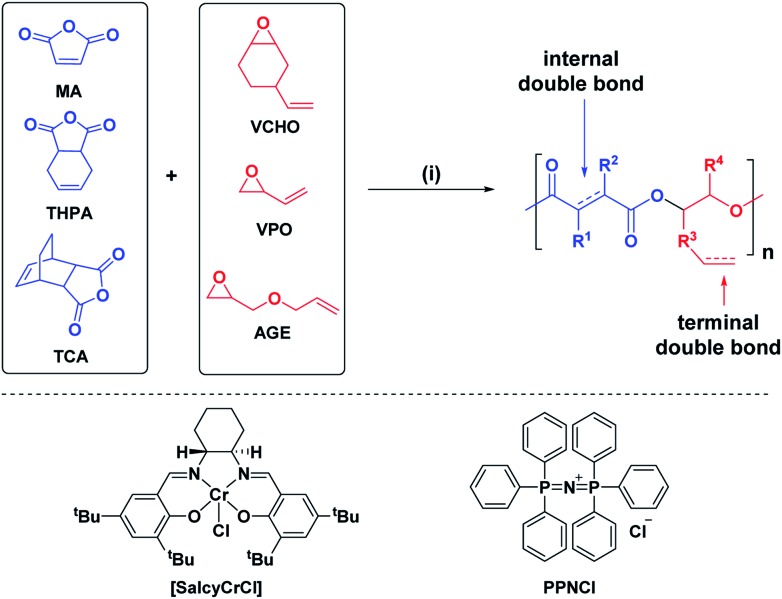
ROCOP of the series of epoxides and anhydrides. Reagents and conditions (i) [SalcyCrCl], PPNCl, 60 °C, toluene, molar ratio: [SalcyCrCl]/PPNCl/CHD/anhydride/epoxide = 1/1/10/100/120, [anhydride] = 5 M.

**Fig. 1:**
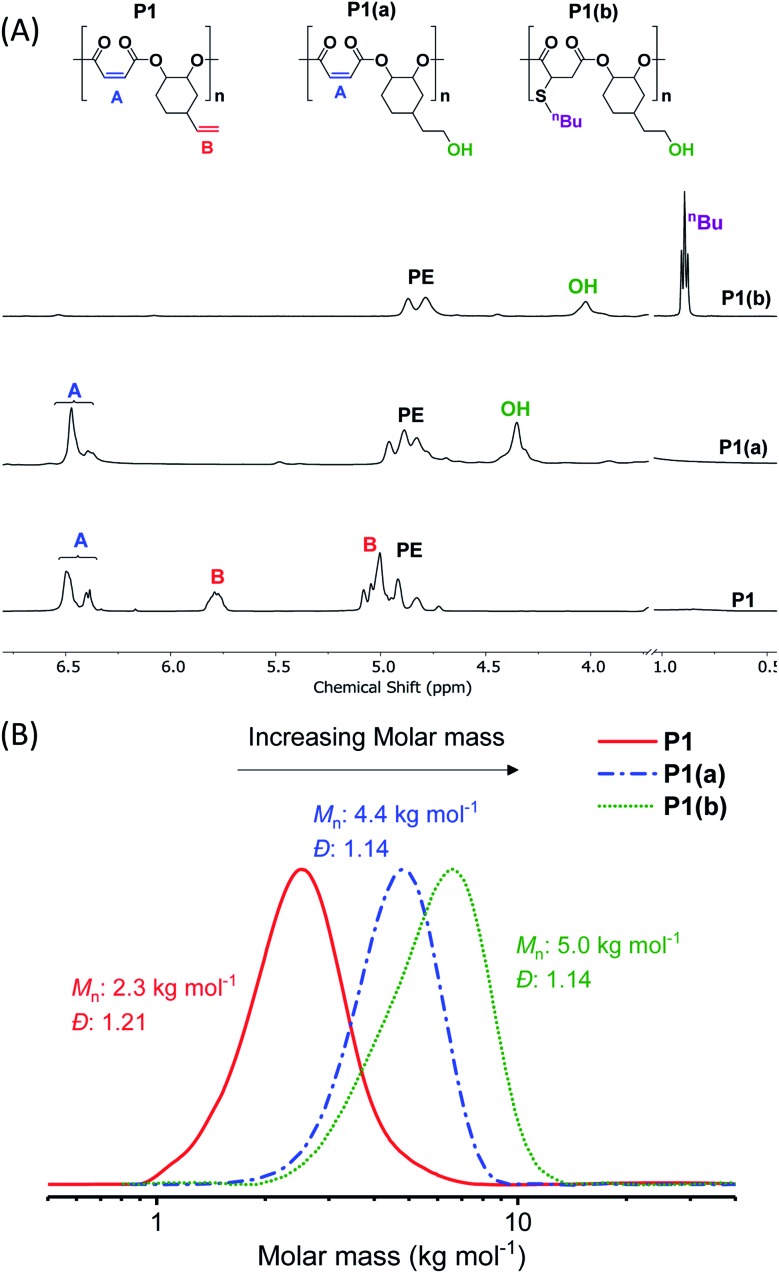
(A) Selected regions of the ^1^H NMR spectra showing the changes in proton resonances of (purified) polymers after functionalization steps (in DMSO-*d*_6_). (B) SEC (in DMF) showing an increase in molar mass after functionalization, in each case with retention of monomodal distributions and narrow dispersity (see ESI† for more details).

The ROCOP reactions were catalysed by a Cr-salen complex, [SalcyCrCl], in combination with an equimolar quantity of bis(triphenylphosphine)iminium chloride (PPNCl) (Scheme 2, see ESI for detailed experimental protocols, Table S1 and Fig. S1†). This catalyst system was selected due to its good activity, substrate scope and selectivity.^21^,^23^,^30^ The catalyst system also results in living polymerizations and is amenable to the use of chain transfer agents. Here, 1,2-cyclohexanediol (henceforth referred to as CHD) is added as the chain transfer agent (10 molar equivalents CHD *vs.* [SalcyCrCl]) to form monomodal molar mass distributions of hydroxyl telechelic chains (Fig. 1B and Table S1†).^29^,^30^,^58^

The crude polymers were isolated by adding the reaction solution, diluted with minimal methylene chloride, dropwise into hexane. The ^1^H NMR spectrum of **P1** exhibits only signals for alternating polyester, there are no detectable ether linkages as evidenced by a lack of signals at 3.00–3.5 ppm (Fig. S2A†). The resonances corresponding to the internal and terminal alkene groups are clearly observed at different chemical shifts: 6.48–6.38 and 5.78 ppm, respectively (peaks ‘A’ and ‘B’ in Fig. 1A (bottom)). When using DMSO-*d*_6_ as the NMR solvent the internal alkene resonance is split into two peaks possibly due to restricted rotation of the vinyl epoxide (Fig. S2A†). The ^1^H–^13^C HSQC and HMBC NMR spectra show these peaks correlate and suggests they are connected to the same carbon atom and correlate with the same carbonyl carbon (Fig. S3B†). The peak splitting is not observed when CDCl_3_ is used as the NMR solvent (Fig. S4†). Nevertheless, DMSO-*d*_6_ is employed in order to properly compare **P1** with its functionalized counterparts (**P1(a)** & **P1(b)**), all of which dissolve in DMSO. The ^13^C{^1^H} NMR spectrum also shows resonances belonging to the maleate and vinyl carbon environments at 141.8 and 130.2 ppm, respectively (Fig. S5†). The MALDI-TOF mass spectrum displays a series of peaks corresponding to hydroxyl-telechelic polyester (α,ω-dihydroxyl terminated), with peaks separated by 222 *m*/*z* as expected for the [VCHO-*alt*-MA] repeating unit (Fig. S6†). Size chromatography (SEC) shows a monomodal peak corresponding to a molar mass of 2.3 kg mol^–1^, with narrow dispersity (*Đ* = 1.21) (Fig. 1B red solid line and Table S3†). Whilst the polyester syntheses were all successful, the copolymerization of VPO/MA ROCOP was noticeably slower and may have led to similar rates of initiation and propagation as evident from the higher *Đ*.

**Fig. 2:**
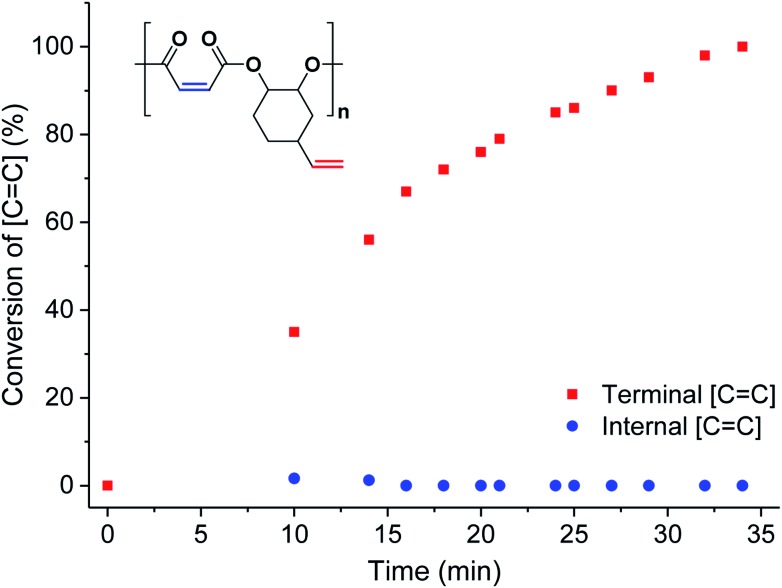
Percentage conversion of the two different alkene groups (internal = blue circles and terminal = red squares) *vs.* time during the hydroboration–oxidation reaction of **P1** (in CDCl_3_, mesitylene as internal standard, see Fig. S7† for stacked NMR spectra).

To investigate the extent of any alkene selectivity during hydroboration–oxidation, the normalized conversions were monitored using *in situ*^1^H NMR spectroscopy (Scheme 1(ii)). The reaction between **P1** and 9-borabicyclo(3.3.1)nonane (9-BBN) ([alkene]/[9-BBN] = 1/1.5) was monitored at 25 °C and using mesitylene as an internal standard (Fig. 2 and S7(LHS)†). The reaction proceeded with high selectivity for the vinyl-alkene substituents resulting in complete consumption of the terminal alkene resonance, at 5.78 ppm, within 40 min. Over this time, the internal alkene, with a diagnostic resonance at 6.48–6.38 ppm, remained unreacted and at the starting concentration (Fig. 2). The selectivity is precisely in line with the earlier alkene hydroboration kinetic investigation which showed that vinyl groups typically react with 9-BBN around 1000 times faster than cyclo-alkenes.^56^ A plot of ln([C=C]_0_/[C=C]) *vs.* time showed a linear fit, illustrating a first-order dependence on the concentration of terminal alkene groups, consistent with the previously proposed hydroboration mechanism (Fig. S7(RHS)†).^57^ All the other polyesters (**P2–P9**) showed equivalent and quantitative selectivity with hydroboration occurring only at the primary alkene groups on the polymer backbone and in a perfectly alternating manner (Fig. S8–S15†).

The hydroborated polymer was reacted with the oxidant, *meta*-chloroperoxybenzoic acid (mCPBA), to produce a polyester which features alternating primary hydroxyl substituents (ethyl alcohol substituents), **P1(a)** (Scheme S1†). Its ^1^H NMR spectrum shows the complete disappearance of signals assigned to the terminal alkene groups and the presence only of alkene resonances corresponding to the internal double bond (peak ‘A’ in Fig. 1A, peak ‘F’ in Fig. S16†). New signals corresponding to the hydroxyl group and its neighbouring methylene protons are seen at 4.36 and 3.42 ppm, respectively (peaks ‘G’ and ‘E’ in Fig. S16†). Importantly, the resonance at 4.87 ppm, assigned to methine carbons adjacent to the polyester backbone, retains the same relative intensity, compared to all other resonances, which confirms that the polymer backbone is not subject to side-reactions in the presence of the oxidant (peak ‘A’ in Fig. S16†). The successful alternating functionalization of **P1** is supported by the SEC data which shows a single monomodal peak at slightly higher molar mass (4.4 kg mol^–1^) than **P1** but with the same narrow dispersity (*Đ* = 1.14) (Fig. 1B).

The alkene-to-hydroxyl transformation was successfully generalized to the whole series of polyesters (**P2–P9**). In most cases, it was highly selective and resulted in quantitative conversion of the primary alkene groups as indicated by a range of characterization techniques, including ^1^H NMR, SEC and DSC (Table S4 and Fig. S17–S22†). In the case of **P4** and **P7**, selective hydroboration was clearly indicated using *in situ*^1^H NMR spectroscopy but the isolation of these hydroxylated polymers was hampered by their high water solubility.

With a successful means to ‘pattern’ the polymer backbones to install hydroxyl substituents at every other repeating unit, the next challenge was to react the remaining internal-alkene monomers to install a second functional group. For the second post-polymerization functionalization, **P1(a)** was reacted with different thiols, using a UV-initiated thiol–ene process, which afforded a series of new (AB)_*n*_ alternating and orthogonally functionalized polyesters, **P1(b)–P1(f)** (Table 1 and Scheme 1).

**Table 1:** Characterization data for orthogonally functionalized alternating polymers^*a*^

#	*T*_g_ ^*b*^ (°C)	Water contact angle^*c*^ (°)	*M*_n_ (kg mol^–1^) [*Đ*]^*d*^
**P1**	70	89 ± 2	2.3 [1.21]
**P1(a)**	93	53 ± 2	4.4 [1.14]
**P1(b)**	69	58 ± 1	5.0 [1.14]
**P1(c)**	78	38 ± 1	5.7 [1.86]
**P1(d)**	86	31 ± 2	10.0 [1.96]
**P1(e)**	8	29 ± 1	3.5 [1.34]
**P1(f)**	–8	46 ± 1	6.0 [1.19]

^*a*^Prepared from **P1a**. Reaction conditions: [DMPA]/[internal alkene]/[thiol] = 1/2.5/10, [alkene]_0_ = 0.25 M in DMSO, UV irradiation (365 nm, 10 W), 25 °C, 1 h.

^*b*^Obtained from DSC (third heating cycle).

^*c*^Reported values are averages obtained from analysis of three different areas of the polymer surface.

^*d*^Measured by SEC (DMF as eluent with 0.075 wt% LiBr, 1 mL min^–1^, 30 °C) calibrated using PMMA standards.

For each of **P1(b)–P1(f)**, the ^1^H NMR spectra show the complete consumption of the maleate alkene protons and indicate quantitative conversion to the appropriate thioether functional groups (Fig. S23†). For instance, the ^1^H NMR spectrum of **P1(b)** shows a new signal at 0.89 ppm assigned to the butyl side-chain substituents (Fig. 1A (top) and S24†). SEC analysis reveals a further slight increase in molar mass (5.0 kg mol^–1^), compared to **P1(a)**, and with retention of monomodal molar mass distributions showing narrow dispersity (*Đ* = 1.14) (Fig. 1B). For polymers **P1(c)–P1(e)**, the molar mass and dispersity values are somewhat larger than expected probably because of interactions between the amine/acidic substituents and the SEC column; these observations are consistent with reports of characterization of similar polymer substituents (Fig. S25†).^59^–^61^

To emphasize the power of this orthogonal functionalization strategy, the control reaction between **P1** and a thiol reagent (1-butanethiol), using otherwise identical reaction conditions, resulted in complete reaction of the all the alkene groups (both vinyl and maleate) and placement of butyl thioethers on every repeating unit (Fig. S26†). Thus, to selectively pattern the polymers requires both hydroboration/oxidation and thiol–ene reactions in the correct order.

The new (AB)_*n*_ functionalized polymers showed quite different properties to the precursor polymers and properties were generally in line with the chemistry of the newly installed substituents. For instance, the addition of the polar hydroxyl substituents to alternating repeating units increased the polymer hydrophilicity. This effect was observed by the reduced water contact angle (by 36 °C) of **P1(a)** compared to **P1** (Table 1 and Fig. S27(a)†). Polymers **P1(c)–P1(f)** feature alternating hydroxyl-polar substituents which all serve to further increase the hydrophilicity compared to **P1(a)**. All the polymers are amorphous as indicated by the only features in the DSC being glass transitions (Fig. S28–S34†). All the hydroxyl functionalized polymers show higher glass transition temperatures than the alkene precursor polyesters, *e.g.***P1(a)**, has a *T*_g_ = 93 °C which is approximately 20 °C higher than **P1** (Fig. S27(b) and S29–S34†). The increase in *T*_g_ is attributed to inter-/intra-chain hydrogen bonding, a phenomenon which is common in biopolymers and has been observed for other hydroxyl-functionalized polymers.^62^,^63^ The entire series of alternating functionalized polymers (**P1(b)–P1(f)**) show lower *T*_g_ values (–8 °C to 86 °C) than **P1(a)** probably due to the increased hydrodynamic volumes (Fig. S28†). For example, **P1(f)** shows a change of >100 °C in *T*_g_ value upon addition of the alternating oligo-ether chains.^40^ The *T*_g_ values of **P1(c)** and **P1(d)** are relatively higher, likely due to neighboring group interactions between the amine and hydroxyl groups.

**Fig. 3:**
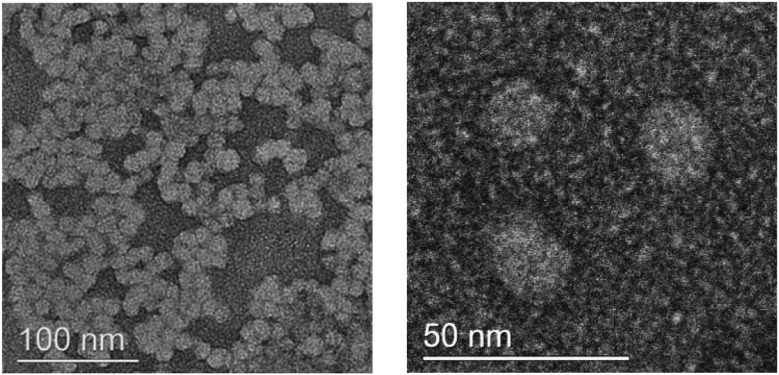
TEM images for the structure of the homopolymer micelle from **P1(b)**.

Most polymer self-assembled nanostructures form from block polymer amphiphiles, *i.e.* featuring hydrophobic and hydrophilic blocks.^64^,^65^ In contrast, there are only limited examples of alternating polymer self-assembly and most of these have applied non-degradable polyacrylate backbones.^4^,^66^–^72^ A guiding principle to observe self-assembly in alternating polymers is that they should feature alternating hydrophobic/philic substituents. **P1(b)** features alternating hydroxyl and butyl substituents and it reproducibly forms stable self-assembled nanostructures in water. This self-assembly is attributed to an adventitious balance of the substituents' hydrophobicity/philicy and the hydrodynamic volumes of chains. Dynamic light scattering (DLS) analysis in water shows that the self-assembled nanostructures have a hydrodynamic diameter of 136.0 ± 1.8 nm (PDI: 0.11) (Fig. S35(a)†). The DLS data shows a low cumulant fit error (<0.005) and the raw correlation data show a flat baseline (Fig. S35(b) and (c)†). Transmission electron microscopy (TEM) analysis reveals that **P1(b)** forms small uniaxial particles with average sizes ∼25 nm (Fig. 3). The apparently large discrepancy in nanoparticle sizes determined by DLS and TEM is tentatively proposed to result from dehydration of the nanoparticles during dry sample preparation for TEM and is consistent with previous reports of alternating polymer self-assemblies.^66^,^72^ The TEM images also showed a lack of hydrophilic/hydrophobic boundary regions suggesting that single chains may adopt conformations so as to maximise the density of polar hydroxyl substituents on the outside of the self-assembled structures and, thus, solvation effects seem to drive self-assembly.^70^,^72^,^73^

In order to investigate whether any of the other amphiphilic polyesters show similar behaviour, **P2(a)** and **P3(a)** were reacted with butanethiol to form **P2(b)** and **P3(b)**. As expected, in each case the complete conversion to thioether was achieved as characterized by ^1^H NMR spectroscopy (Fig. S36 and S37†). SEC analysis of the amphiphiles shows monomodal molar mass distributions and the data are consistent with no significant polyester backbone degradation/side-reactions (Table S3†). Both **P2(b)** and **P3(b)** also self-assembled in water, with DLS measurements showing hydrodynamic diameters of 381.0 ± 1.8 nm (PDI: 0.211) and 302.7 ± 2.0 nm (PDI: 0.08), respectively (Fig. S38†). The samples were also each analyzed by TEM, which showed spherical nanostructures with diameters more closely aligned with those obtained by DLS (*ca.* 300 nm and *ca.* 250 nm for **P2(b)** and **P3(b)**, respectively) (Fig. S39 and S40†). We tentatively ascribe the differences between hydrodynamic diameters obtained by TEM and DLS for **P1(b)** compared to **P2(b)**/**P3(b)**, to either drying effects during TEM sample preparation or to different self-assembly structures. Future investigations should continue to explore the self-assembled structures of these alternating and degradable polymers.

## Conclusion

In summary, a series of orthogonally functionalized alternating polyesters were prepared exploiting the high AB monomer sequence selectivity of epoxide/anhydride ring-opening copolymerization. Starting from polyesters featuring alternating terminal and internal alkene substituents, a hydroboration–oxidation allows for selective and quantitative conversion of the vinyl substituents into hydroxyl groups, leaving the adjacent internal alkenes unreacted. Subsequently, the remaining internal alkene substituents were selectively and quantitatively transformed into thioether groups featuring alkyl, oligoether, amine and carboxylic acid substituents. This method is particularly useful as a generalizable, scalable and efficient route to many different AB alternating functionalized polyesters; the majority of which could not be accessed by other routes. These functionalized AB polyesters are an important addition to the sustainable polymer palette and are particularly relevant as they allow for straightforward tuning of chain glass transition temperature and hydrophilicity. Future work will exploit the precision substituent separations and use the materials to investigate adjacency, cooperativity and multi-valent effects. Precision placement of hydroxyl substituents is expected to significantly influence both hydrolysis and biodegradation rates, particularly those dependent on serine proteases. In the future, such polyesters, sequentially patterned with hydrophilic, -phobic and ionisable groups, could be useful as bio-inspired antimicrobials, degradable cell penetrating polymers and to improve upon the properties of biodegradable plastics.

## Conflicts of interest

There are no conflicts of interest to declare.

## Supplementary Material

Supplementary information
