# Supplementary material for: Orthogonal functionalization of alternating polyesters: selective patterning of (AB)n sequences
Source: Chem Sci. 2019 Sep 20;10(43):9974–80. doi: 10.1039/c9sc03756j (PMC6968736; doi:10.1039/c9sc03756j)
Supplement: Supplementary file 1 [file SC-010-C9SC03756J-s001.pdf]

Electronic Supplementary Material (ESI) for Chemical Science.
This journal is © The Royal Society of Chemistry 2019

Electronic Supplementary Information for

Orthogonal Functionalization of Alternating Polyesters: Selective Patterning of $(AB)_n$ Sequences

Ni Yi,[a†] Thomas T. D. Chen,[a†] Junjuda Unruangsri,[b] Yunqing Zhu,[b] and Charlotte K. Williams[a*]

[a] Chemistry research laboratory, Department of Chemistry, University of Oxford, 12 Mansfield Road, Oxford, OX1 3TA, UK
[b] Department of Chemistry, Imperial College London, London, SW7 2AZ, UK
[†]Joint first authors

charlotte.williams@chem.ox.ac.uk

Table of Contents

Section, Scheme, Table or Figure	Pages
Experimental Section	3-6
**Figure S1.** Structures of the alternating polyesters **P1**-**P9**, all featuring internal and terminal alkene functionalities.	4
**Table S1.** Composition and Properties of **P1**-**P9**.	5
**Scheme S1.** One-pot hydroboration-oxidation reaction to convert the polyester terminal alkenes to alternating hydroxyl groups.	5
**Scheme S2.** Photo-initiated thiol-ene reactions to transform the hydroxyl-functionalized alternating polyesters into orthogonally functionalized (AB)n Polyesters	6
**Table S2**. Preparation Data for Polymers **P1(b)** - **P1(f)** and data to accompany in Table **1**.	6
**Figure S2.** NMR spectra for **P1**.	7
**Figure S3.** 2D NMR spectra for **P1**.	8
**Figure S4.** ^{1}H NMR spectrum of polymer **P1** ($CDCl_3$, 500.0 MHz, 298 K).	9
**Figure S5.** $^{13}C\{^{1}H\}$ NMR spectrum of **P1** (*d*$_6$-DMSO, 500.0 MHz, 298 K).	9
**Figure S6.** MALDI-ToF spectrum of **P1** synthesized with CHD as the CTA, (Table S1, #1).	10
**Table S3.** SEC characterization data (in THF and DMF) for **P1**, **P2** and **P3**.	10
**Figure S7.** NMR and kinetic data illustrating selective terminal alkene hydroboration reaction.	11
**Figure S8**. Conversion vs. Time Plot for alkene groups (internal and terminal) for **P2** during the hydroboration-oxidation reaction.	11
**Figure S9.** Conversion vs. Time Plot for alkene groups (internal and terminal) for **P3** during the hydroboration-oxidation reaction.	11
**Figure S10.** Conversion vs. Time Plot for alkene groups (internal and terminal) for **P4** during the hydroboration-oxidation reaction.	12
**Figure S11.** Conversion vs. Time Plot for alkene groups (internal and terminal) for **P5** during the hydroboration-oxidation reaction.	12
**Figure S12.** Conversion vs. Time Plot for alkene groups (internal and terminal) for **P6** during the hydroboration-oxidation reaction.	12

**Figure S13.** Conversion vs. Time Plot for alkene groups (internal and terminal) for **P7** during the hydroboration-oxidation reaction.	13
**Figure S14.** Conversion vs. Time Plot for alkene groups (internal and terminal) for **P8** during the hydroboration-oxidation reaction.	13
**Figure S15.** Conversion vs. Time Plot for alkene groups (internal and terminal) for **P9** during the hydroboration-oxidation reaction.	13
**Figure S16.** ^{1}H NMR spectrum of **P1(a)** (DMSO-d_6, 400 MHz, 298 K).	14
**Table S4.** Characterization Data for Hydroxyl-functionalized polyesters **P1(a)** - **P9(a)**.	14
**Figure S17.** ^{1}H NMR spectrum of **P2(a)** ($CDCl_3$, 400 MHz, 298 K).	15
**Figure S18.** ^{1}H NMR spectrum of **P3(a)** ($CDCl_3$, 400 MHz, 298 K).	15
**Figure S19.** ^{1}H NMR spectrum of **P5(a)** (d_6-DMSO, 400 MHz, 298 K).	16
**Figure S20.** ^{1}H NMR spectrum of **P6(a)** (d_6-DMSO, 400 MHz, 298 K).	16
**Figure S21.** ^{1}H NMR spectrum of **P8(a)** (d_6-DMSO, 400 MHz, 298 K).	17
**Figure S22.** ^{1}H NMR spectrum of **P9(a)** ($CDCl_3$, 400 MHz, 298 K).	17
**Figure S23**. Stacked ^{1}H NMR spectra of **P1(c)-P1(f)** showing the disappearance of the internal alkene resonances after thiol-ene reactions (d_6-DMSO, 500.0 MHz, 298 K).	18
**Figure S24**. ^{1}H NMR spectrum of **P1(b)** (d_6-DMSO, 500.0 MHz, 353 K).	18
**Figure S25**. Stacked SEC traces of **P1(b)-P1(f)**.	19
**Figure S26**. ^{1}H NMR data illustrating the non-selective nature of UV-initiated thiol-ene reactions.	19
**Figure S27.** (a) Photographs of water droplets with their measured water contact angles of **P1** (top) and **P1(a)** (bottom). (b) DSC thermograms of **P1** and **P1(a)**, showing an increase in T_g after functionlization.	20
**Figure S28.** (A) Photographs of water droplets on polymer- coated glass surfaces (**P1(b)-P1(f)**). (B) Overlaid DSC traces of polymers **P1(b)-P1(f)**.	21
**Figure S29**. DSC thermograms of **P2**, **P2(a**) **and P2(b)**, showing the changes in T_g after functionalization.	22
**Figure S30.** DSC thermograms of **P3**, **P3(a)** and **P3(b),** showing the changes in T_g after functionalization.	22
**Figure S31**. DSC thermograms of **P5** and **P5(a),** showing the change in T_g after functionalization.	22
**Figure S32**. DSC thermograms of **P6** and **P6(a),** showing the change in T_g after functionalization.	23
**Figure S33.** DSC thermograms of **P8** and **P8(a),** showing the change in T_g after functionalization.	23
**Figure S34.** DSC thermograms of **P9** and **P9(a),** showing the change in T_g after functionalization.	23
**Figure S35.** Dynamic light scattering data collected from an aqueous solution of **P1(b)** (10 $mgmL^{-1}$).	24
**Figure S36**. ^{1}H NMR spectrum of **P2(b)** ($CDCl_3$, 500.0 MHz, 298 K).	25
**Figure S37**. ^{1}H NMR spectrum of **P3(b)** ($CDCl_3$, 500.0 MHz, 298 K).	25
**Figure S38**. DLS data for self-assembly of **P2(b)** (left) and **P3(b)** (right).	26
**Figure S39**. TEM images for the structure of the homopolymer micelle from **P2(b)**.	26
**Figure S40.** TEM images for the structure of the homopolymer micelle from **P3(b).**	26

Materials and Methods

Materials. All manipulations were carried out using standard Schlenk line or dry-box techniques under an inert atmosphere of nitrogen. All glassware were dried at 160 °C for 20 h and cooled under vacuum prior to use. Solvents were dried by passing through a column of appropriate drying agent, degassed and stored under nitrogen atmosphere. Chloroform-d_3 was dried over CaH_2, distilled under reduced pressure and stored over 4 Å molecular sieves, under nitrogen atmosphere.

All solvents and reagents were obtained from commercial sources (Alfar Aesar, VWR International and Sigma-Aldrich) and used as received unless stated otherwise. Vinyl cyclohexene oxide (vCHO) was purchased from Acros, and fractionally distilled from CaH_2. Cyclohexene diol was recrystallised from ethyl acetate and stored under nitrogen atmosphere.

NMR. ^{1}H, ^{13}C and 2D NMR spectra were recorded using Bruker AV400 or AVD500 MHz spectrometer at ambient temperature (unless stated otherwise). ^{1}H, ^{13}C{^{1}H} NMR spectra were referenced internally to residue protio-solvent (^{1}H) or solvent (^{13}C) resonances, and are reported relative to tetramethylsilan. Mestrenova software (version 8.0) was used to process and analyse the spectra.

SEC. The polymers were dissolved in SEC grade THF and filtered through a 0.2 µm syringe filter prior to analysis. SEC data were determined by a Shimadzu LC-20AD instrument using MALLS detector (Wyatt Dawn 8+), with THF as the eluent, at a flow rate of 1.0 mL/min at 30 °C. Two Mixed Bed PSS SDV linear S columns were used in series. The MALLS detector was calibrated by polystyrene standard. For functional polymers, a different Agilent SEC instrument was used to characterize the molecular weights and dispersities, with PSS GRAM columns in series using DMF (+0.075% w/v LiBr) as the eluent. Retention times were normalised using water as a flow rate marker. Molecular weights were calculated relative to a set of narrow polystyrene standards.

MALDI-ToF mass spectrometry. MALDI-ToF MS experiments were carried out on Waters/Micromass MALDI micro MX spectrometer, using a dithranol matrix in THF at loading of 1:1 with potassium trifluoroacetate (KO_2CCF_3) as the cationizing agent. Elemental Analyses were carried out by the Elemental Analysis Service at London Metropolitan University.

Thermal properties test. The thermal properties were measured using DSC Q2000 (TA Instruments, UK). A sealed empty crucible was used as a reference, and the DSC was calibrated using indium. Samples were heated from room temperature to 125 °C, at a rate of 10 °C $\cdot$min^{-1}, under helium flow, and were kept at 125 °C for 2 min to erase the thermal history. Subsequently, the samples were cooled to -100 °C, at a rate of 10 °C $\cdot$min^{-1}, and kept at -100 °C $\cdot$min^{-1} for further 2 mins, followed by a heating procedure from -100 °C to 130 °C, at a rate of 10 °C $\cdot$min^{-1}. Each sample was run for three heating–cooling cycles. The glass transition temperatures (T_g) reported are taken from the third cycle.

Water contact angle measurements. Static water contact angles were measured using a Drop Shape Analysis System (EasyDrop, Krüss, Germany). A 30 µL drop of ultra-pure water (MilliQ water, Millipore, MA, USA) was placed on the polymer film surfaces and static water

contact angle was measured. The measurements were performed on three different areas of each slide and the values were averaged.

DLS. The DLS measurements were conducted using a Zetasizer Nano series instrument (Malvern Instrument Zen 1600). The scattering angle was set at 173° and all the aqueous aggregate solutions were put into disposable PMMA cuvettes for analysis.

Zeta potential. The physical stability of the self-assembled nanostructures is characterized by the zeta potential using a Zetasizer Nano series instrument (Malvern Instruments). All determinations were repeated for three times and the obtained values were averaged.

TEM. Amorphous carbon-coated copper TEM grids (Agar Scentific) were plasma glow-discharged for 15 s to generate a hydrophilic surface. Individual samples (2-4 µL) were placed onto the freshly discharged grids for 5 mins, before being blotted with a filter paper to remove excess solutions. Uranium acetate (0.5 w/v %) was used to stain the sample by placing onto the sample-loaded grids for 5 mins and then removing the excessive staining solution. The grids were dried in fume hood overnight prior to characterization. TEM images were obtained by Tianyi Chen on JEOL 3000F scanning transmission electron microscope, equipped with an Oxford X-Max 80 SDD EDX detector, at an operating voltage of 200 kV.

Ring opening copolymerization of epoxides/anhydride

Typically, copolymerizations of epoxides and anhydrides were carried out according to the following procedure. In a glovebox, [salenCr^{III}Cl] catalyst, **1**, (10.0 mg, 16.0 µmol), Bis(triphenylphosphoranylidene)ammonium chloride (PPNCl) (9.0 mg, 16 µmol), cyclohexene diol (18.0 mg, 0.2 mmol), anhydride (1.6 mmol) and epoxide (1.90 mmol) were dissolved in toluene (2.0 mL) placed in a Schlenk tube (molar ratio: **1**/PPNCl/CHD/anhydride/epoxide = 1/1/10/100/120). The reaction mixture was stirred at 60 °C for a predetermined time period. The volatiles were removed *in vacuo*. The crude polymer was dissolved in THF, filtered through an Amberlyst-15 column and purified by repetitive precipitation from hexane (3 x 100 mL). The purified polymer was then collected was then dried *in vacuo*.

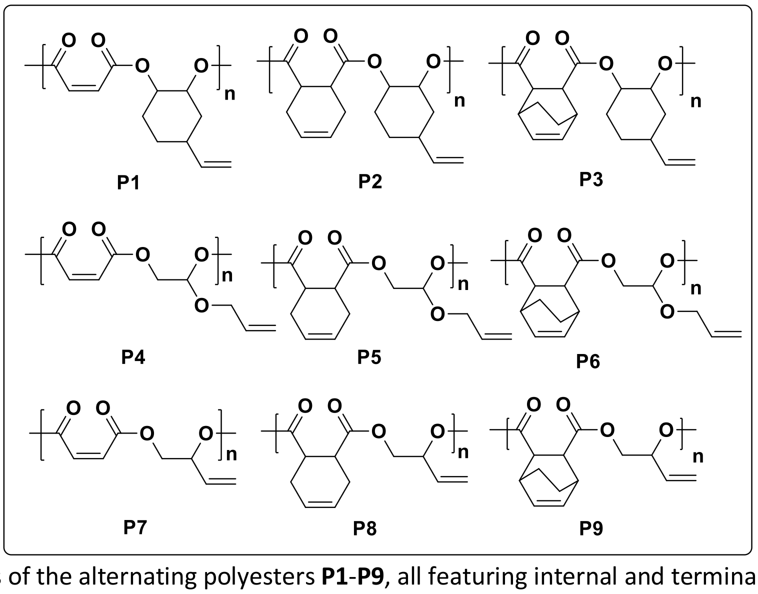

Figure S1. Structures of the alternating polyesters **P1**-**P9**, all featuring internal and terminal alkene functionalities.

Table S1. Composition and Properties of **P1-P9**.

#	Epoxide	Anhydride	Time (h)	Conv. [%][a]	M_n ($kgmol^{-1}$) [Đ][b]	T_g (°C)[c]
**P1**	VCHO	MA	6	>99	1.7 [1.13]	70
**P2**	VCHO	THPA	15	80	2.9 [1.11]	80
**P3**	VCHO	CHMA	15	>99	5.6 [1.16]	117
**P4**	VPO	MA	46	80	1.5 [1.30]	-27
**P5**	VPO	THPA	33	90	3.3 [1.18]	14
**P6**	VPO	CHMA	19	>99	2.9 [1.18]	56
**P7**	AGE	MA	12	>99	4.9 [1.13]	-28
**P8**	AGE	THPA	6	>99	3.2 [1.18]	1
**P9**	AGE	CHMA	3.5	>99	3.6 [1.14]	23

Polymerization conditions: 60 °C, molar ratio: 1/PPNCl/CHD/anhydride/epoxide = 1/1/10/100/120, [anhydride] = 5M in toluene. [a]conversions were obtained from ^1H NMR spectra [b]M_n and $Đ$ were measured by SEC (THF as eluent, 1 mL/min, 30 °C) calibrated using polystyrene standards. [c]T_g were obtained from DSC (third heating cycle)

Hydroboration-oxidation of polyesters

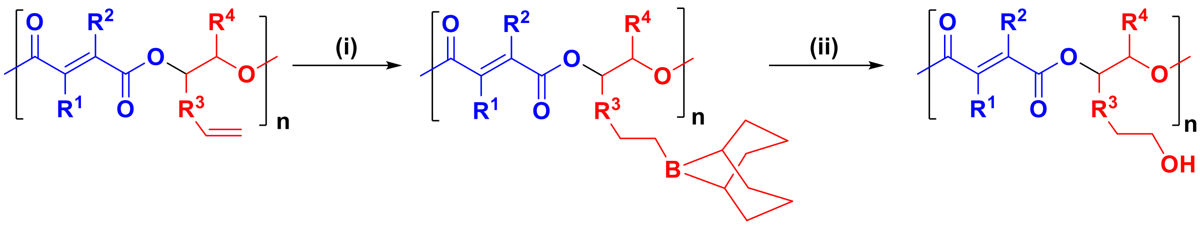

Scheme S1. One-pot hydroboration-oxidation reaction to convert the polyester terminal alkenes to alternating hydroxyl groups. (i): 9-BBN dimer, 1.5 h, 25 °C, THF (ii): mCPBA, 2 h, 25 °C, THF, molar ratio: $[alkene]_o$/[9-BBN]/[mCPBA] = 1/1.5/5.1), $[alkene]_o$ = 0.03 M in THF

To a solution of the polyester (0.60 mmol) in THF (20 mL), was added a solution of 9-borabicyclo(3.3.1)nonane (9-BBN) dimer (2.41 g, 9.90 mmol) in THF (20.0 mL). After stirring the reaction mixture for 2 h at 25 °C, 0.1 mL of pure methanol was added to react with any 9-BBN left after hydroboration. The oxidation of the hydroborated polymer was carried out in the same Schlenk flask by adding meta-chloroperoxybenzoic acid (mCPBA) (11.62 g, 0.07 mol) at 0 °C under an inert atmosphere. The relative molar ratio of $[C{=}C]_0/[9\text{-}BBN]_0/[m\text{-}CPBA]_0$ was 1/1.5/5.1. After stirring the solution mixture at 25 °C for 2 h, a methanol/water (10:1 v/v, 10 mL) mixture was added dropwise. The volatiles were removed under reduced pressure to ~2 mL. The product was precipitated from a 1 M $NaHCO_3$ aqueous solution (500 mL), washed with water and dried *in vacuo* for 72 h.

NMR scale reaction between polyester and 9-BBN.

To a solution of the polyester (0.008 mmol) (in $CDCl_3$ (0.30 mL) in an NMR tube, equipped with a J. Young Teflon valve, was added a solution of 9-BBN dimer (0.02 g, 0.088 mmol) in $CDCl_3$ (0.30 mL) at 25 °C. The reaction was monitored for 2 h using ^1H NMR spectroscopy with mesitylene as an internal standard.

Thiol-ene 'Click' reaction between P1(a) and functional thiols.

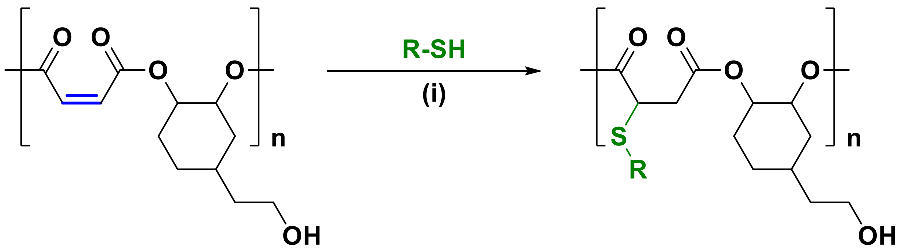

Scheme S2. Photo-initiated thiol-ene reactions to transform the hydroxyl-functionalized alternating polyesters into orthogonally functionalized (AB)n Polyesters. (i): DMPA, thiol reagent, DMSO, irradiated under UV (365 nm, 10W) for 2 h, molar ratio: [DMPA]/[alkene]$_0$/[thiol] = 1/2.5/10, [alkene]$_0$ = 0.25 M in DMSO

Typically, **P1(a)**, 2,2-Dimethoxy-2-phenylacetophenone (DMPA) and thiols were dissolved in DMSO (1.7 mL), resulting in the molar ratio of [C=C]$_0$/[thiol]$_0$/[DMPA]$_0$ = 1/2/0.4. The freeze-pump-thaw procedure was conducted for three cycles to deoxygenate the solution. Then, the thiol-ene reaction was induced by UV irradiation (365 nm, 10W) at 25 °C for 1 h. The crude reaction mixture was dried *in vacuo* to remove the solvent. The final polymer was purified by repetitive precipitation from diethyl ether.

Table S2. Preparation Data for Polymers **P1(b)** - **P1(f)** and data to accompany in Table **1**.

#	Thiol	Loadings P1(a)	Loadings DMPA	Loadings Thiol
**P1(b)**	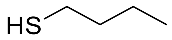	100.0 mg	42.0 mg	89.0 µL
**P1(c)**	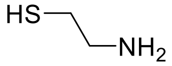	100.0 mg	42.0 mg	94.0 mg
**P1(d)**	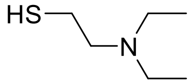	100.0 mg	42.0 mg	140.0 mg
**P1(e)**	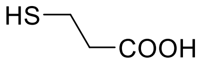	100.0 mg	42.0 mg	72.0 µL
**P1(f)**	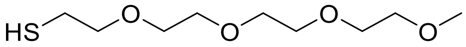	100.0 mg	42.0 mg	186.0 mg

All polymerizations were run in DMSO as the reaction solvent, under UV irradiation (365 nm, 10 W) at 25 °C for 1 h, with an initial concentration of [C=C] at 0.25 M.

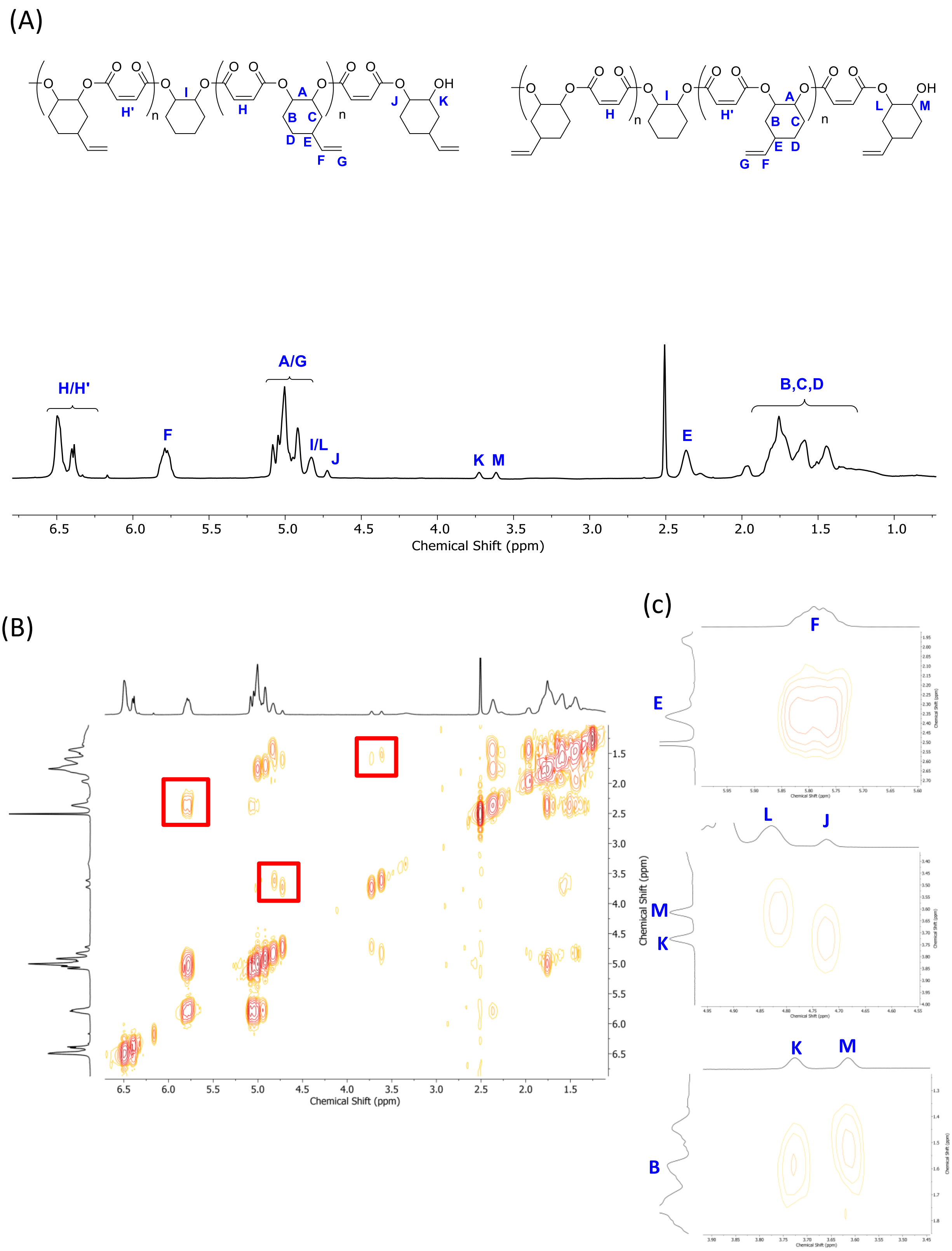

Figure S2. NMR spectra for **P1**. (A) ^{1}H NMR spectrum of polymer **P1** (d_6-DMSO, 500.0 MHz, 298 K) (B) ^{1}H-^{1}H COSY NMR spectrum of polymer **P1**. (d_6-DMSO, 500.0 MHz, 298 K) (C) Enlarged ^{1}H-^{1}H COSY NMR spectra corresponding to the selected area in (B). The signals at (5.76, 2.35) is assigned to the correlation between vinyl groups and the adjacent methine protons. The signals at (4.81, 3.62) and (4.72, 3.72) are assigned to the correlations between the end group methylene protons. The signals at (3.73, 1.59) and (3.61, 1.54) are assigned to the correlation between end group and backbone methylene protons.

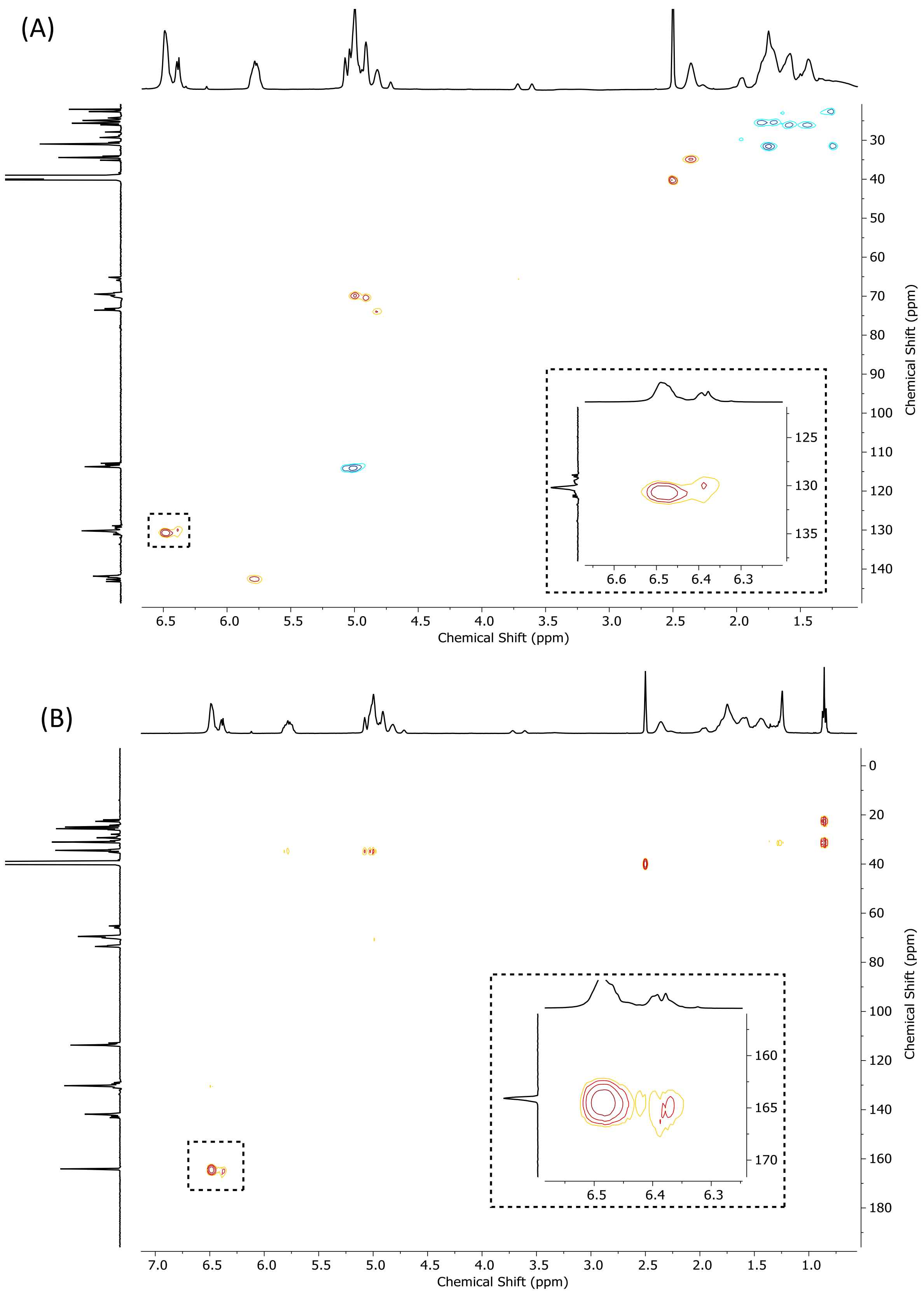

Figure S3. 2D NMR spectra for **P1**. (A) ^{1}H-^{13}C HSQC NMR spectrum of **P1** (d_6-DMSO, 500.0 MHz, 298 K). Insert: magnified region showing the peaks at 6.48-6.38 ppm (internal alkenes) correlating to similar ^{13}C environment. (B) ^{1}H-^{13}C HMBC NMR spectrum of **P1** (d_6-DMSO, 500.0 MHz, 298 K). Insert: magnified region showing peaks at 6.48-6.38 ppm (internal alkenes) correlating to the carbonyl carbon.

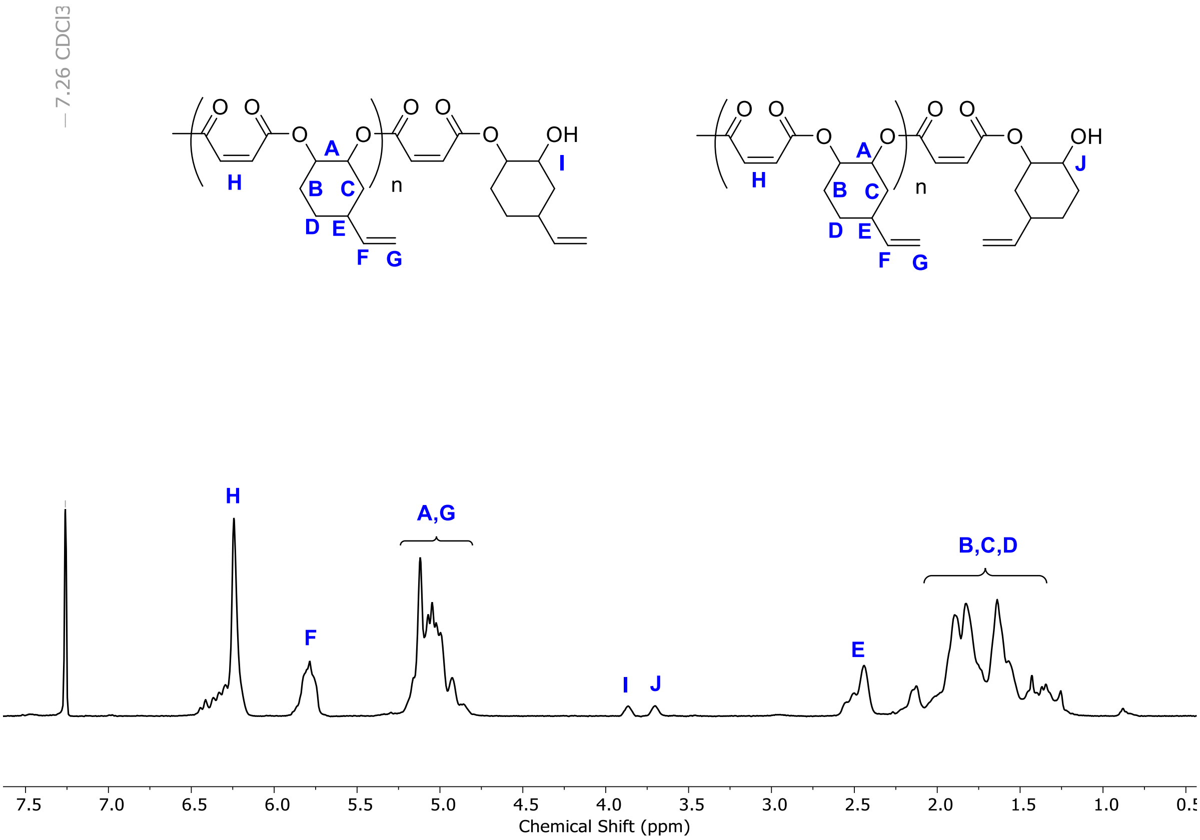

Figure S4. ^{1}H NMR spectrum of polymer **P1** ($CDCl_3$, 500.0 MHz, 298 K).

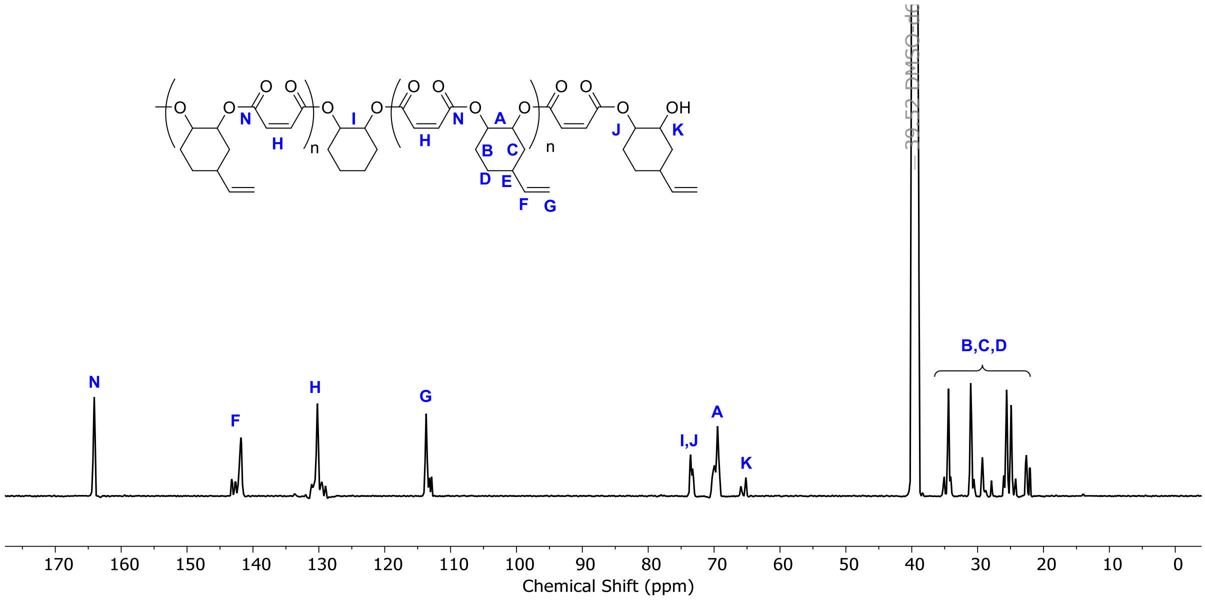

Figure S5. $^{13}C\{^{1}H\}$ NMR spectrum of **P1** (d_6-DMSO, 500.0 MHz, 298 K).

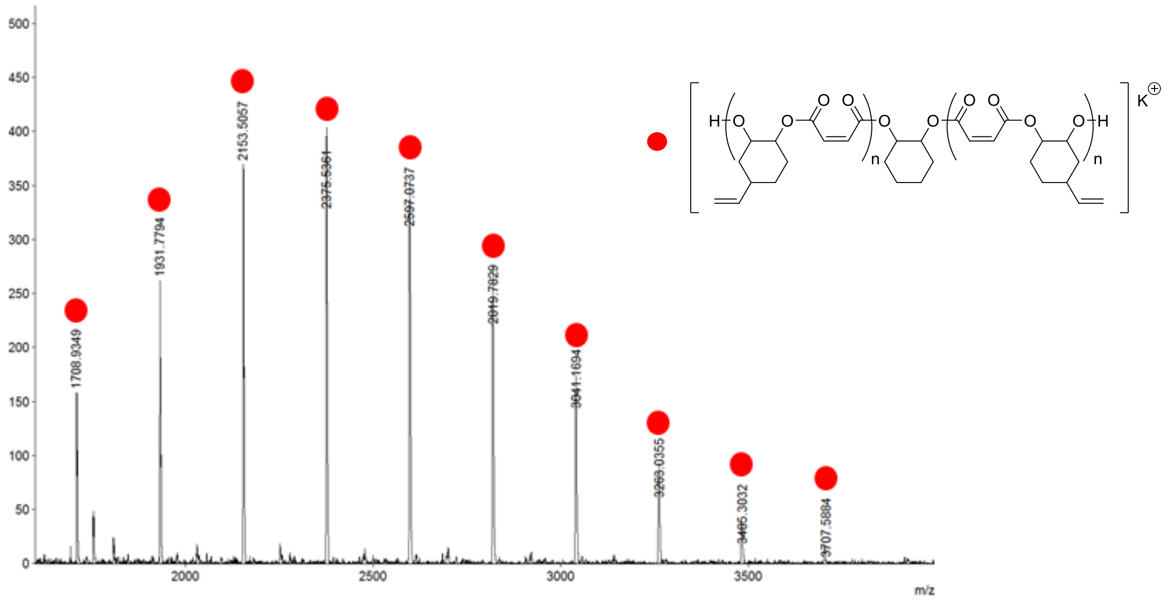

Figure S6. MALDI-ToF spectrum of **P1** synthesized with CHD as the CTA, (Table S1, #1). The polyester series show m/z = [116.16(cyclohexanediol) + (222.24 x n) (MA-*alt*-vCHO) + 39.1 (K^+)].

Table S3. SEC characterization data (in THF and DMF) for **P1**, **P2** and **P3**.

**Polyester**	**THF GPC** (M_n (kg mol^{-1}) [$Đ$])			**DMF GPC** (M_n (kg mol^{-1}) [$Đ$])		
	Precursor	Hydroxyl functionalized (a)	Dual-functionalized (b)	Precursor	Hydroxyl functionalized (a)	Dual-functionalized (b)
**P1**	1.7 [1.13]	N/A*	N/A*	2.3 [1.21]	4.4 [1.14]	5.0 [1.14]
**P2**	2.9 [1.21]	2.2 [1.22]	4.0 [1.24]	2.4 [1.20]	4.5 [1.27]	5.6 [1.27]
**P3**	6.0 [1.16]	5.6 [1.18]	6.2 [1.13]	4.4 [1.13]	5.8 [1.26]	7.0 [1.24]

*Polymer not soluble in solvent

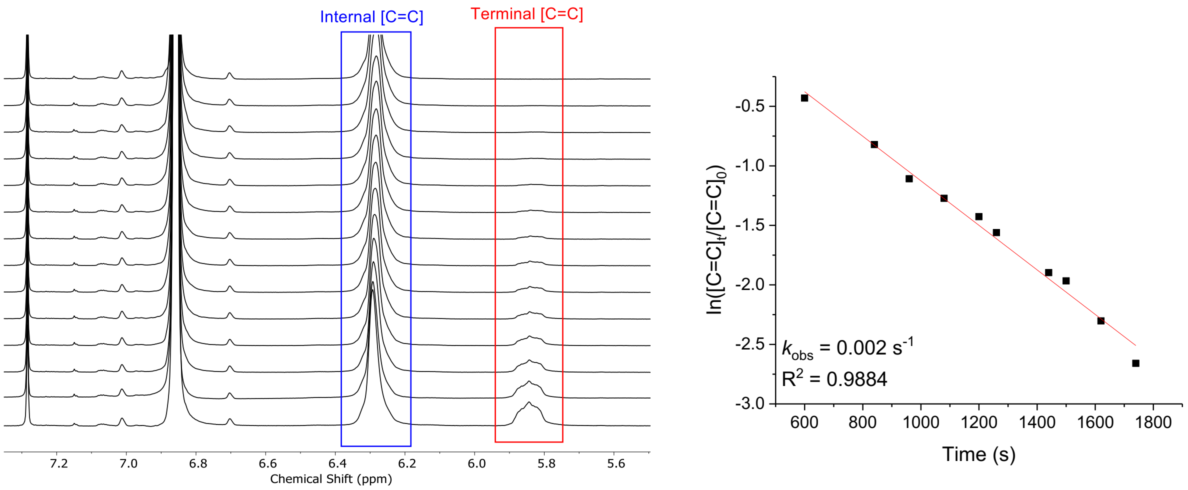

Figure S7. NMR and kinetic data illustrating selective terminal alkene hydroboration reaction. (LHS) Changes in the ^{1}H NMR resonance of alkene groups during the reaction of 1 with 9-BBN ($CDCl_3$, with mesitylene (10 equiv., 6.86 ppm) as an internal standard). (RHS) Plot of ln($[C=C]_0/[C=C]_t$) vs time of terminal alkene groups. Rate = $k[C=C]^1[9\text{-BBN}]^x$, $k_{obs} = k[9\text{-BBN}]^x$.

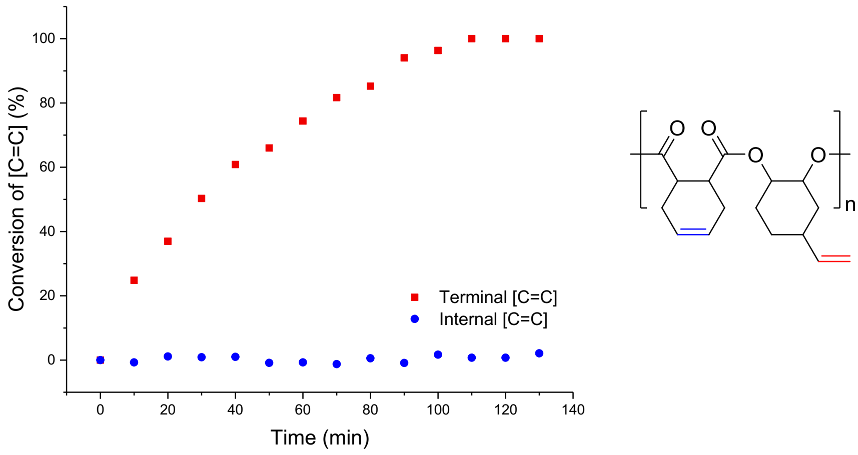

Figure S8. Conversion vs. Time Plot for alkene groups (internal and terminal) for **P2** during the hydroboration-oxidation reaction.

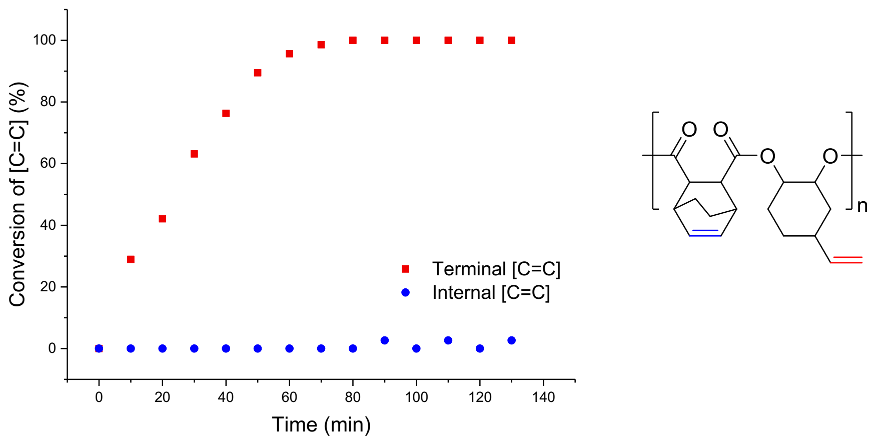

Figure S9. Conversion vs. Time Plot for alkene groups (internal and terminal) for **P3** during the hydroboration-oxidation reaction.

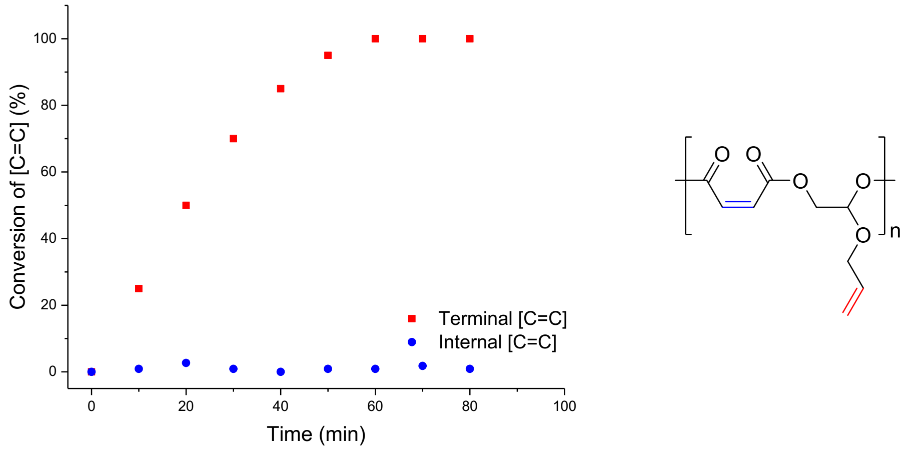

Figure S10. Conversion vs. Time Plot for alkene groups (internal and terminal) for **P4** during the hydroboration-oxidation reaction.

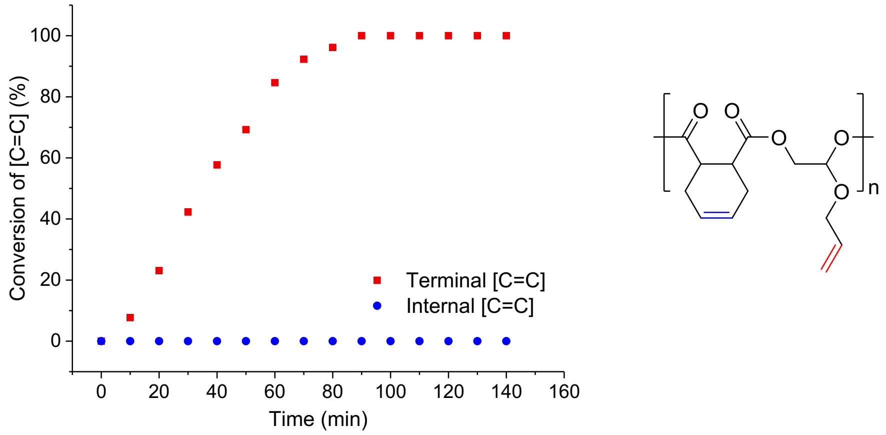

Figure S11. Conversion vs. Time Plot for alkene groups (internal and terminal) for **P5** during the hydroboration-oxidation reaction.

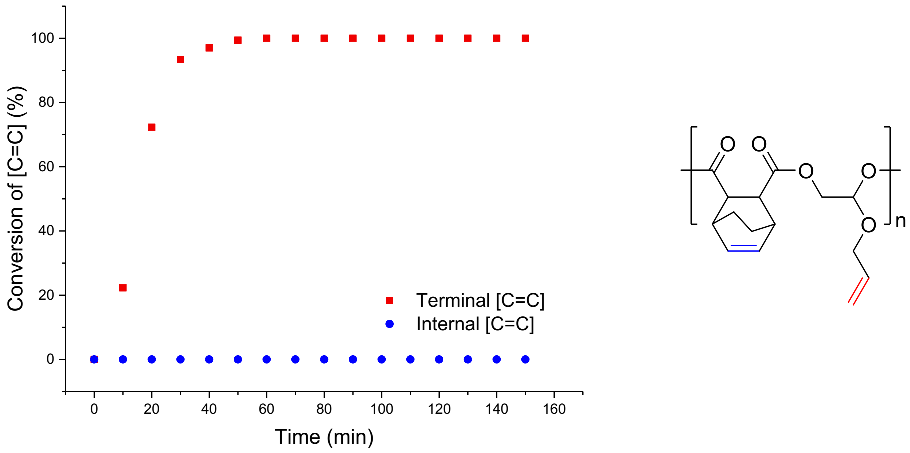

Figure S12. Conversion vs. Time Plot for alkene groups (internal and terminal) for **P6** during the hydroboration-oxidation reaction.

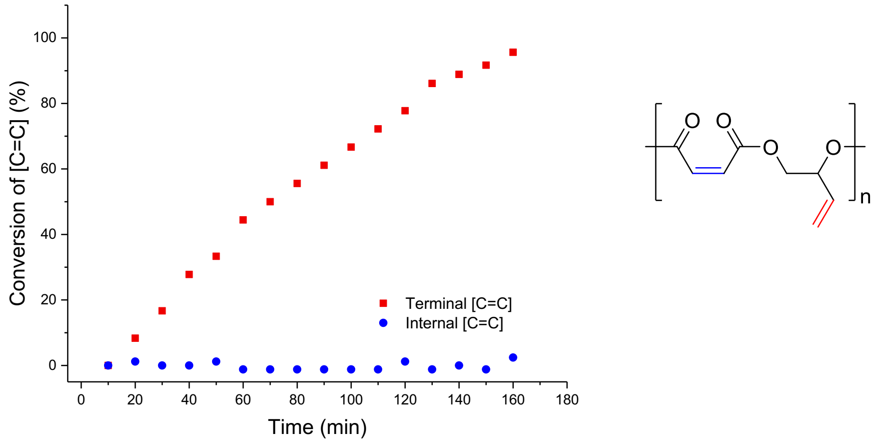

Figure S13. Conversion vs. Time Plot for alkene groups (internal and terminal) for **P7** during the hydroboration-oxidation reaction.

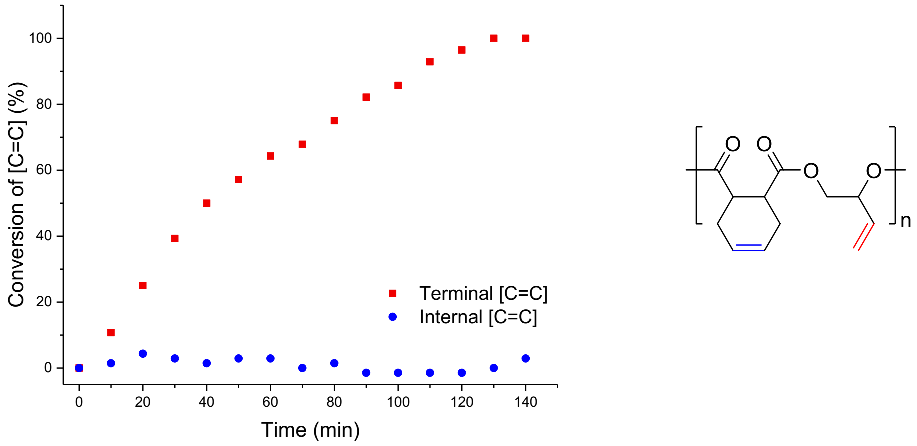

Figure S14. Conversion vs. Time Plot for alkene groups (internal and terminal) for **P8** during the hydroboration-oxidation reaction.

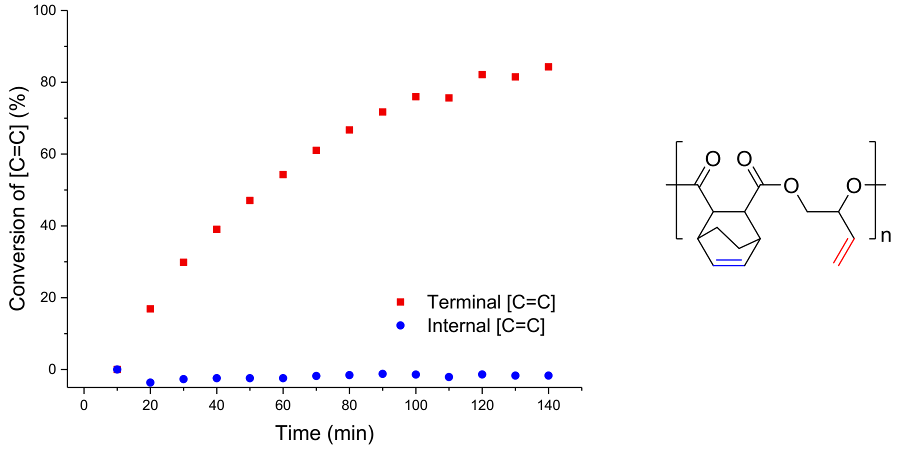

Figure S15. Conversion vs. Time Plot for alkene groups (internal and terminal) for **P9** during the hydroboration-oxidation reaction.

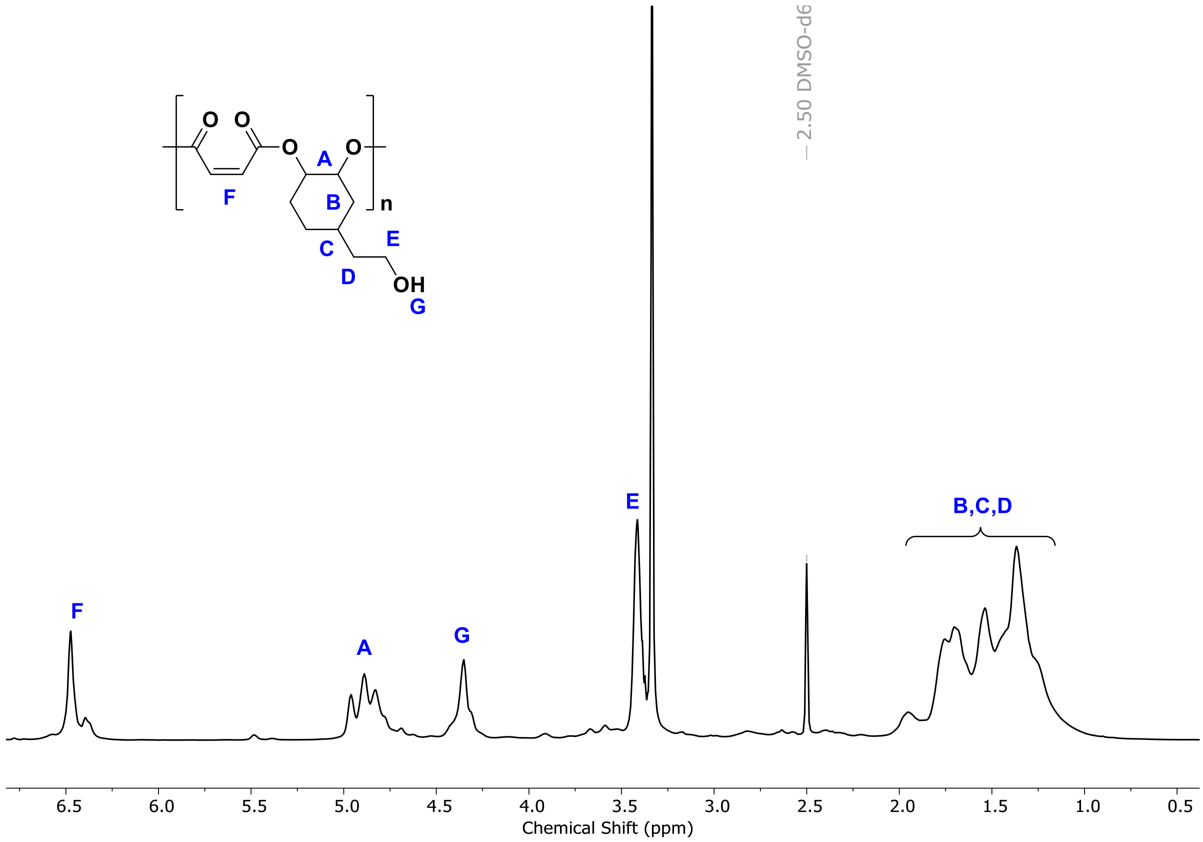

Figure S16. ^{1}H NMR spectrum of **P1(a)** (DMSO-d_6, 400 MHz, 298 K).

Table S4. Characterization Data for Hydroxyl-functionalized polyesters **P1(a)** - **P9(a)**.

#	Epoxide	Anhydride	M_n[a]	$Đ$[a]	T_g[b]
**P1(a)**	VCHO	MA	4.4	1.14	98
**P2(a)**	VCHO	THPA	4.9	1.12	106
**P3(a)**	VCHO	CHMA	6.0	1.18	133
**P5(a)**	VPO	THPA	3.0	1.31	55
**P6(a)**	VPO	CHMA	4.2	1.11	75
**P8(a)**	AGE	THPA	3.8	1.19	28
**P9(a)**	AGE	CHMA	5.6	1.15	41

[a]M_n and $Đ$ were measured by SEC (DMF as eluent with 0.075 wt% LiBr, 1 mL/min, 30 °C) calibrated using PMMA standards. [b]T_g were obtained from DSC (third heating cycle)

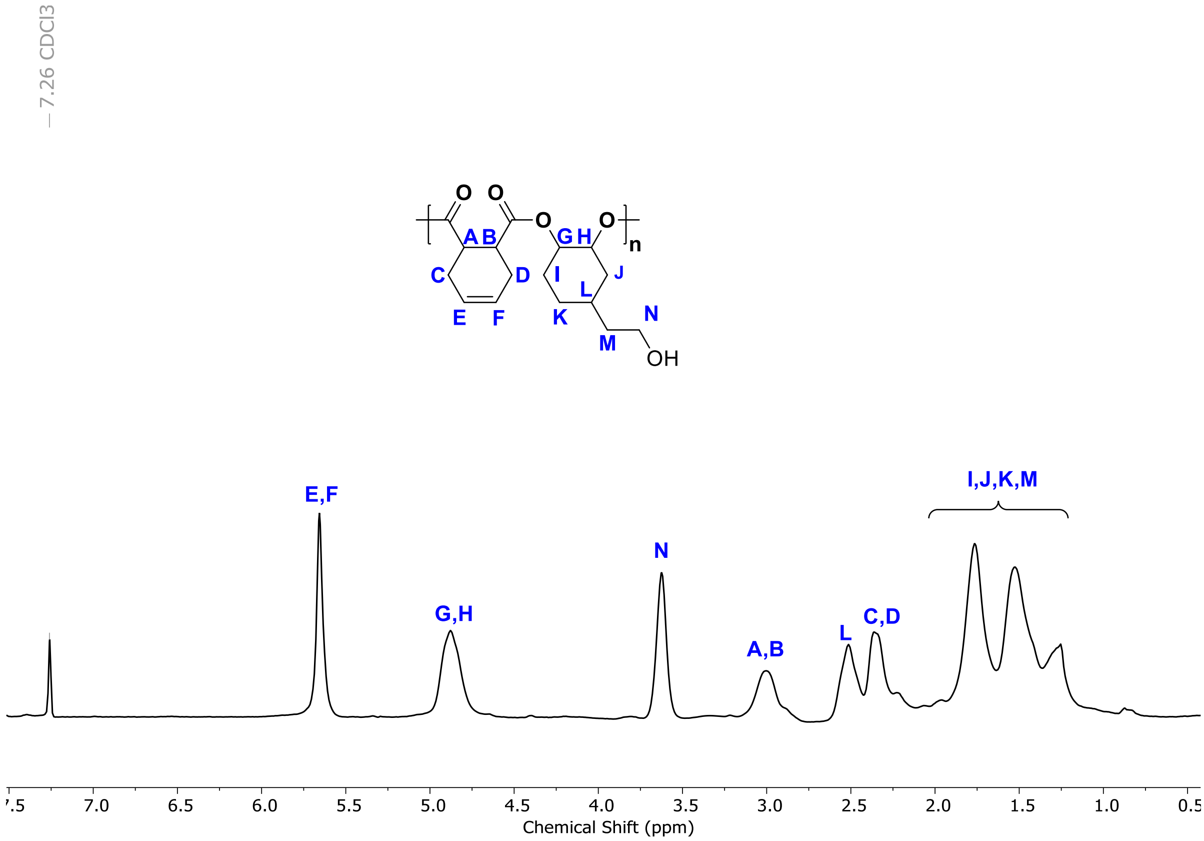

Figure S17. ^{1}H NMR spectrum of **P2(a)** ($CDCl_3$, 400 MHz, 298 K).

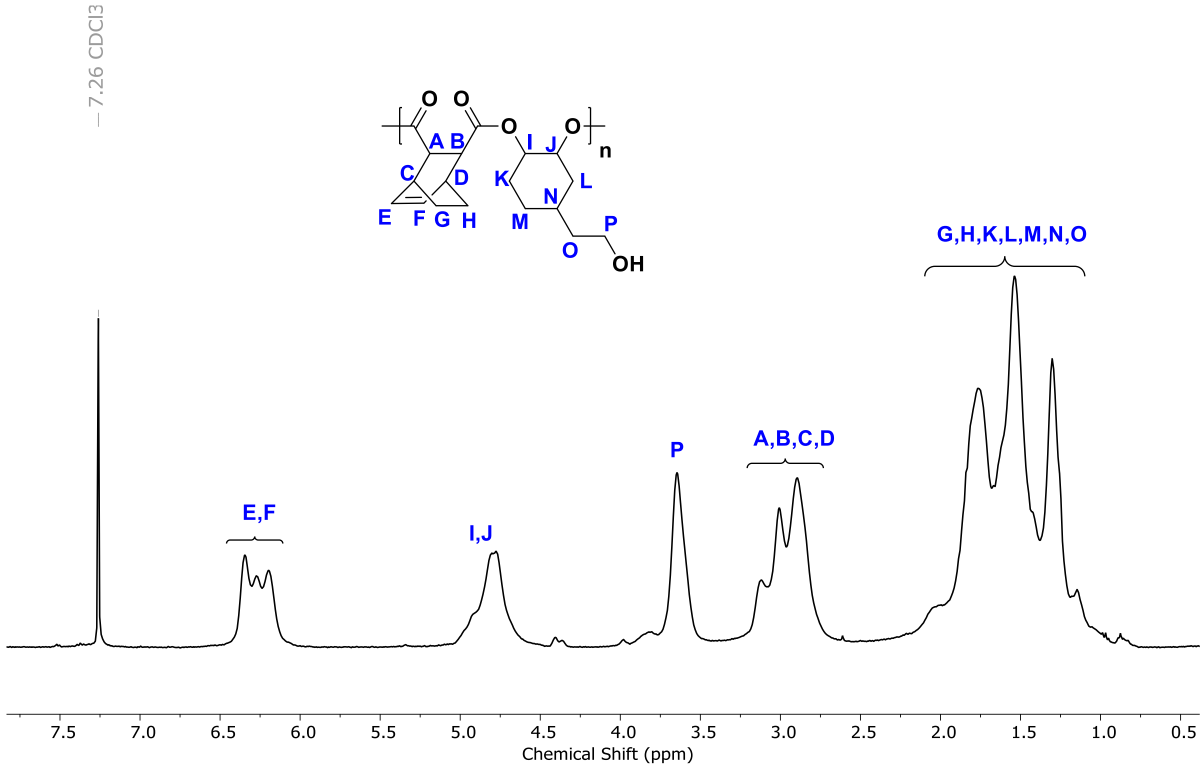

Figure S18. ^{1}H NMR spectrum of **P3(a)** ($CDCl_3$, 400 MHz, 298 K).

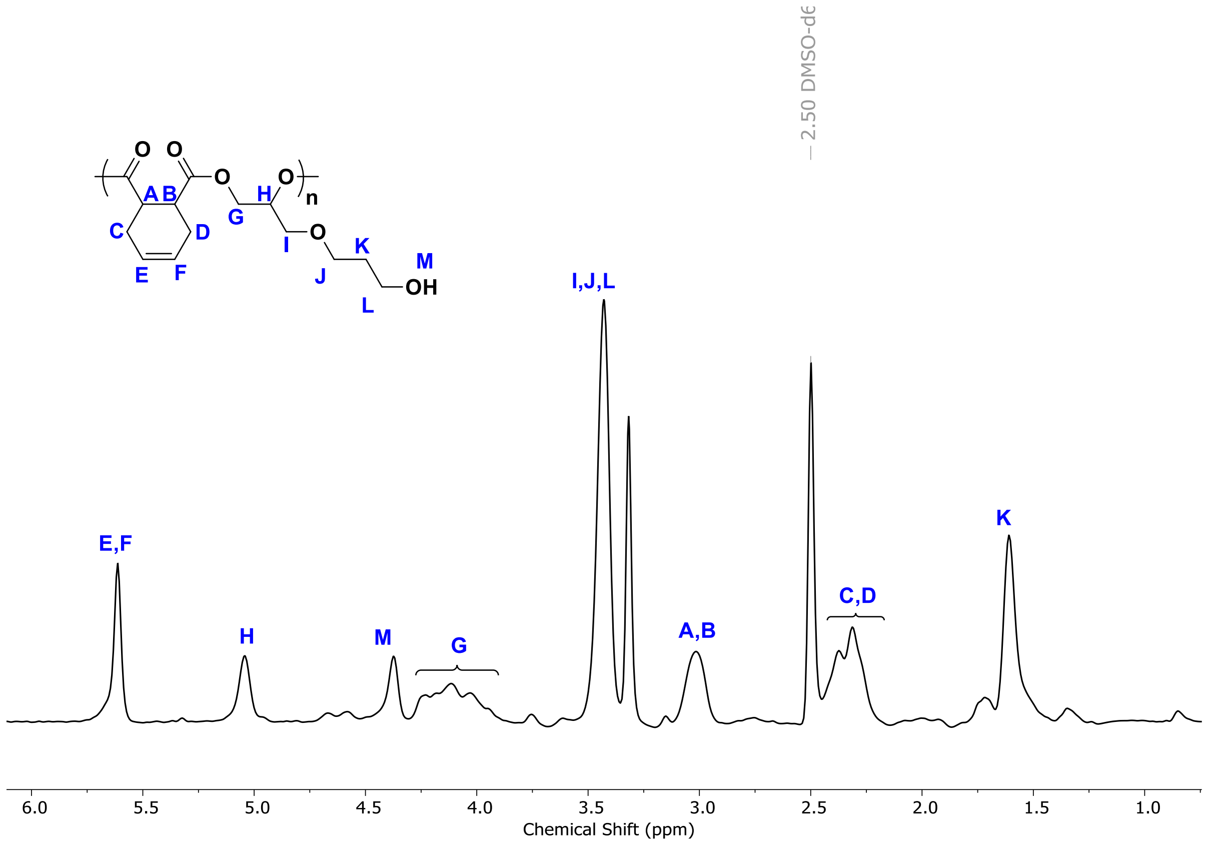

Figure S19. ^{1}H NMR spectrum of **P5(a)** (d_6-DMSO, 400 MHz, 298 K).

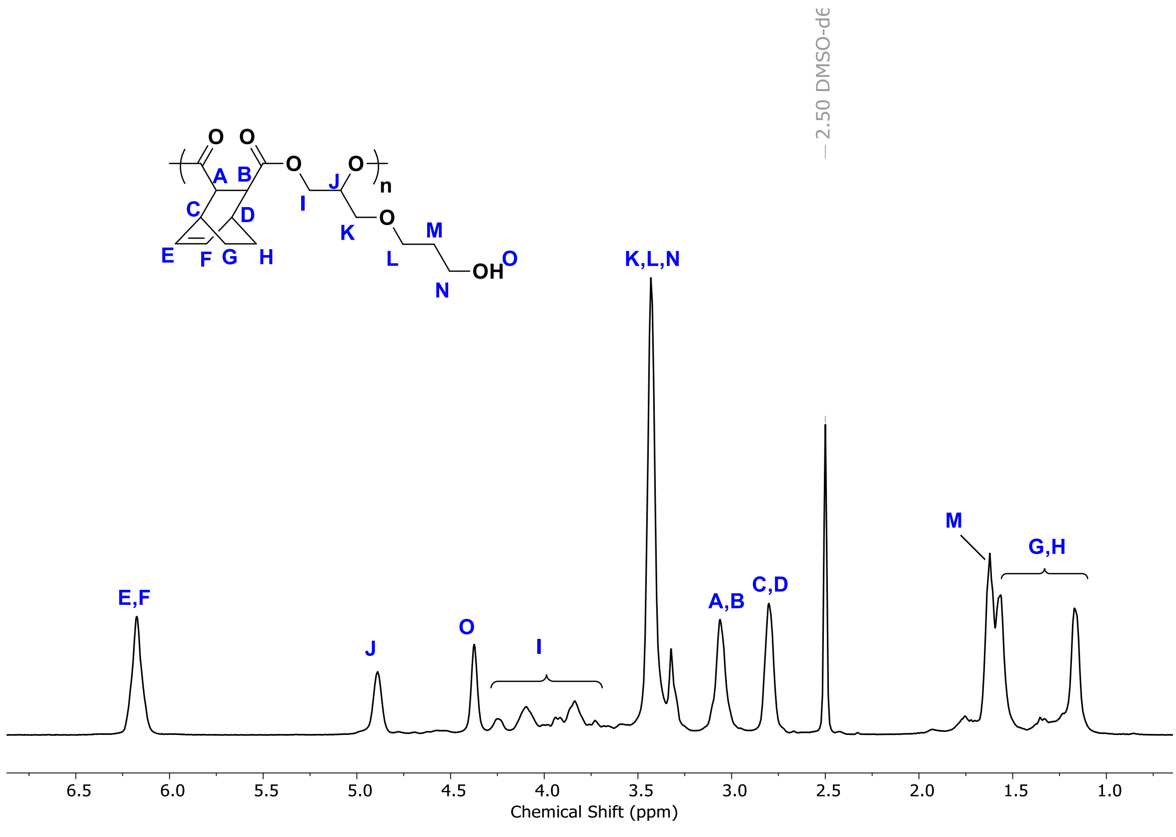

Figure S20. ^{1}H NMR spectrum of **P6(a)** (d_6-DMSO, 400 MHz, 298 K).

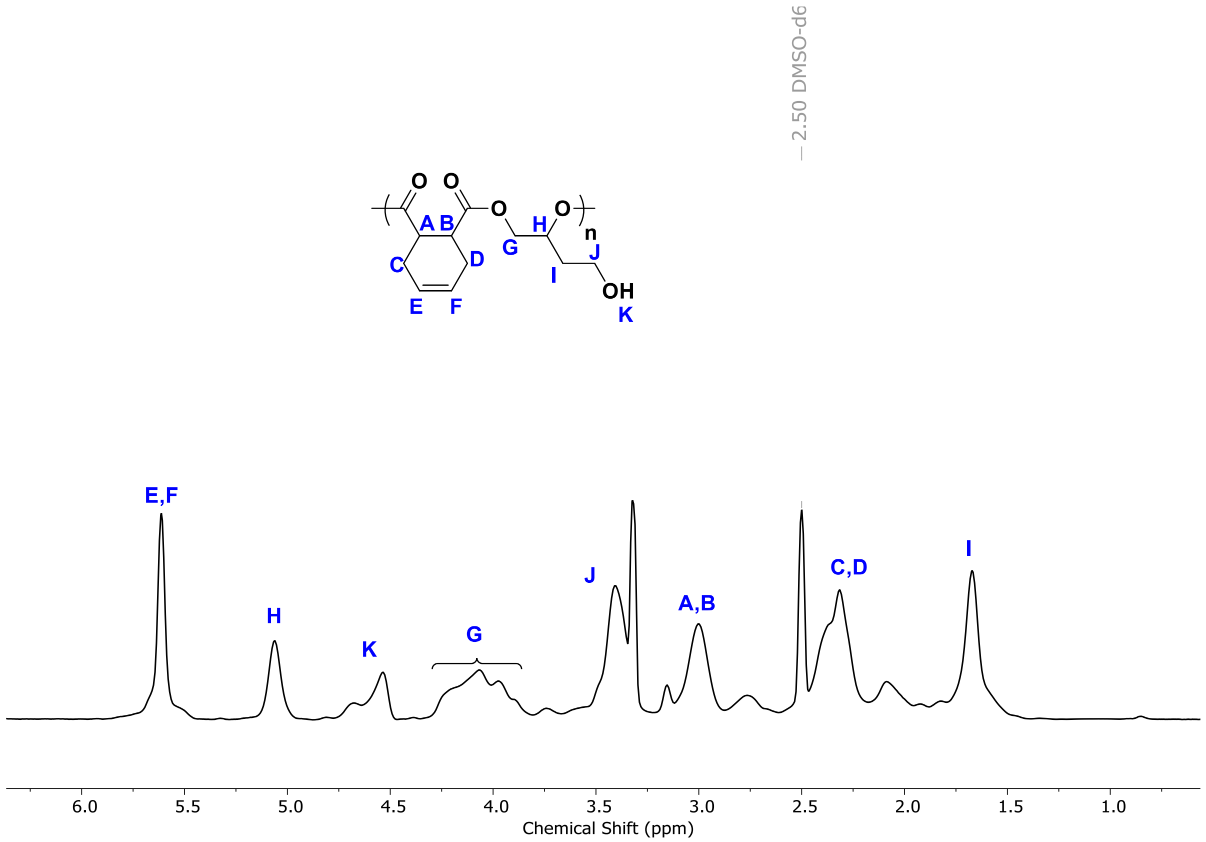

Figure S21. ^{1}H NMR spectrum of **P8(a)** (d_6-DMSO, 400 MHz, 298 K).

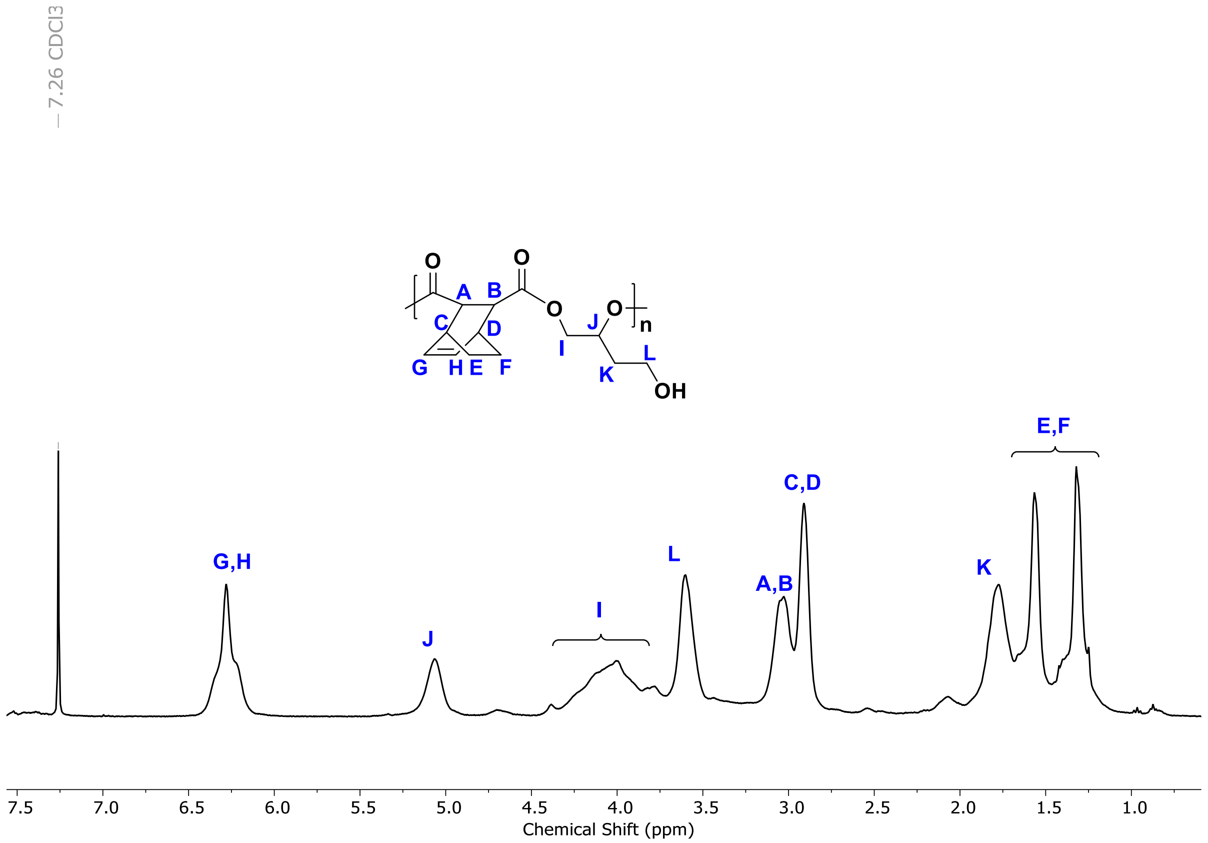

Figure S22. ^{1}H NMR spectrum of **P9(a)** ($CDCl_3$, 400 MHz, 298 K).

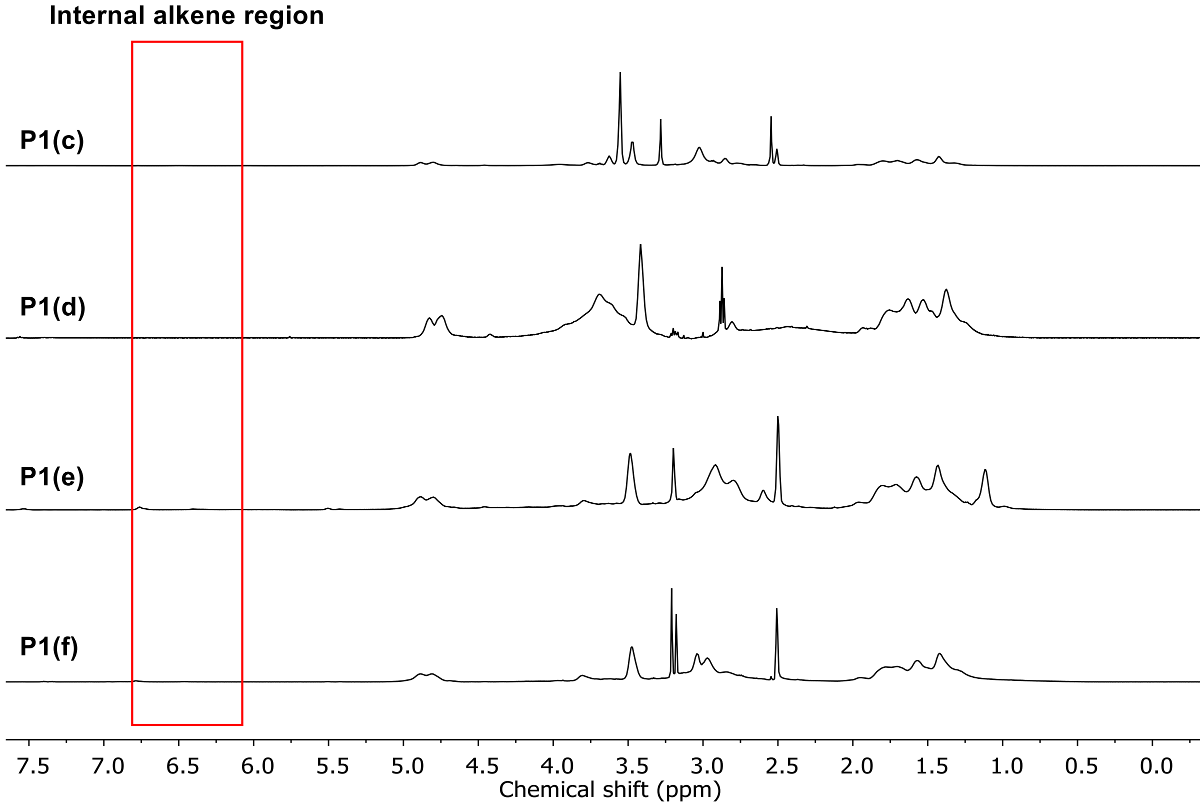

Figure S23. Stacked ^{1}H NMR spectra of **P1(c)-P1(f)** showing the disappearance of the internal alkene resonances after thiol-ene reactions (d_6-DMSO, 500.0 MHz, 298 K).

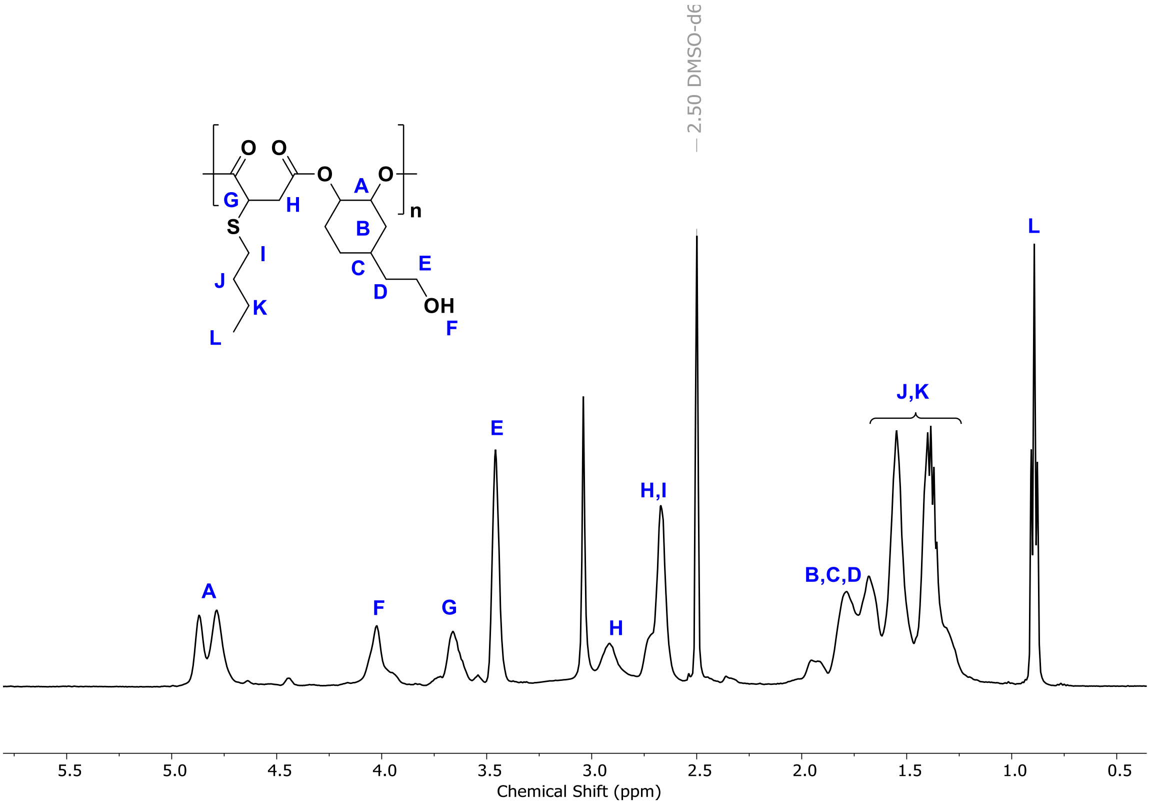

Figure S24. ^{1}H NMR spectrum of **P1(b)** (d_6-DMSO, 500.0 MHz, 353 K).

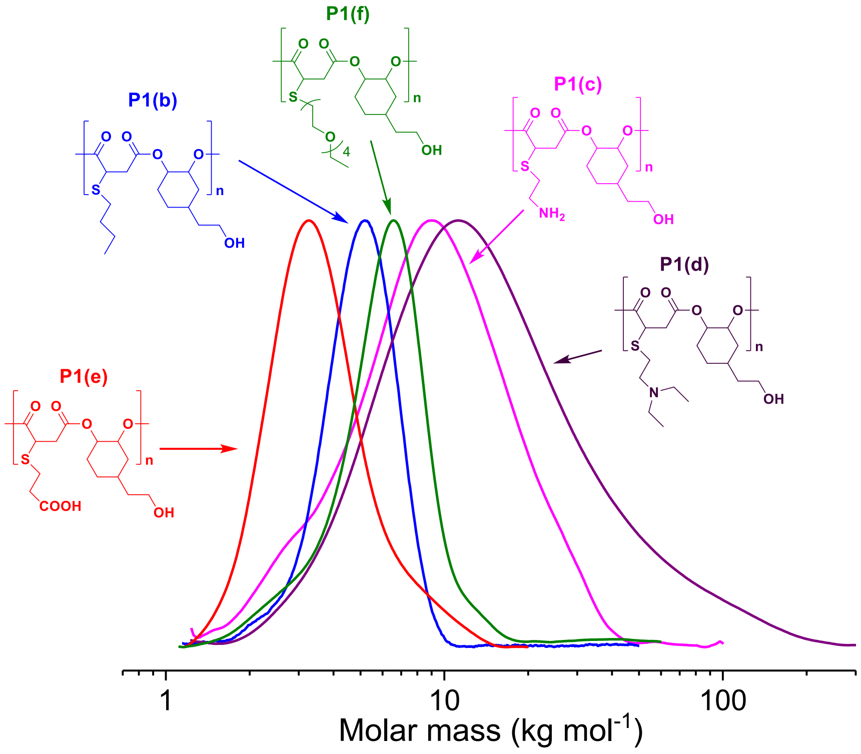

Figure S25. Stacked SEC traces of **P1(b)-P1(f)**.

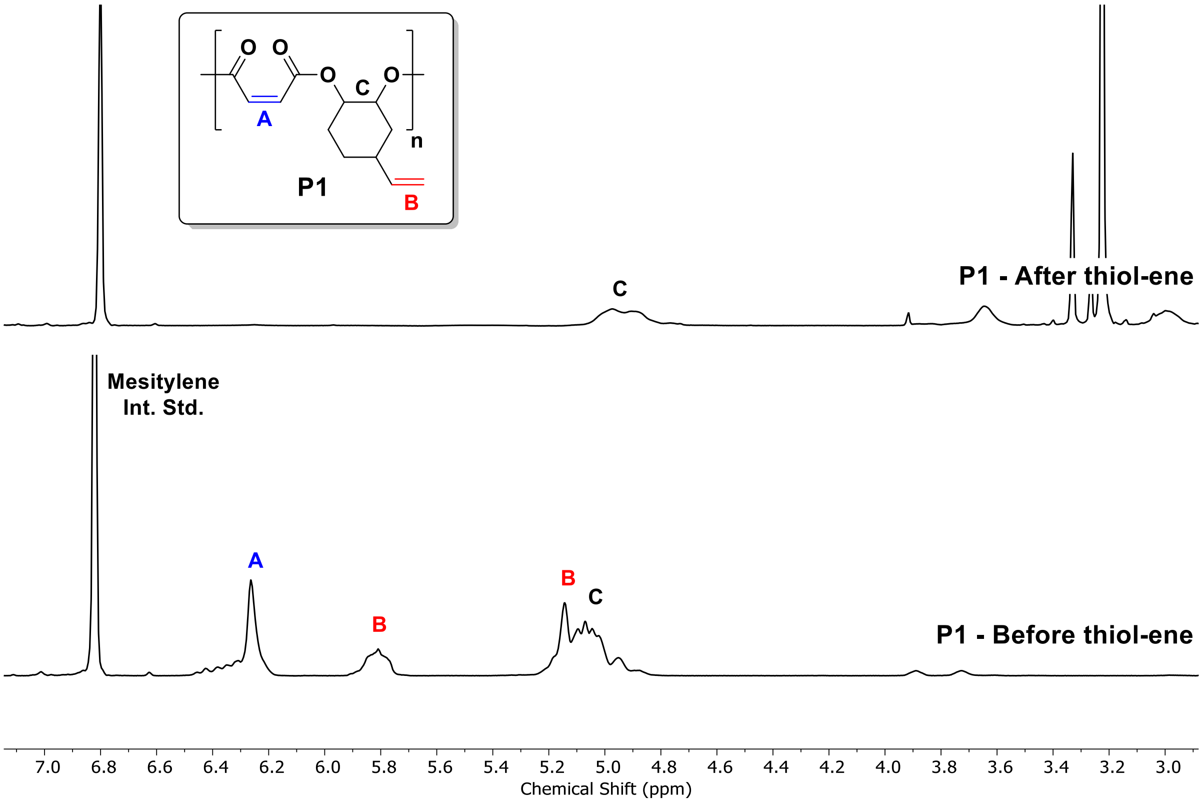

Figure S26. ^{1}H NMR data illustrating the non-selective nature of UV-initiated thiol-ene reactions. (Bottom) **P1** before application of thiol-ene reaction. (Top) after the thiol-ene reaction (mesitylene as internal standard).

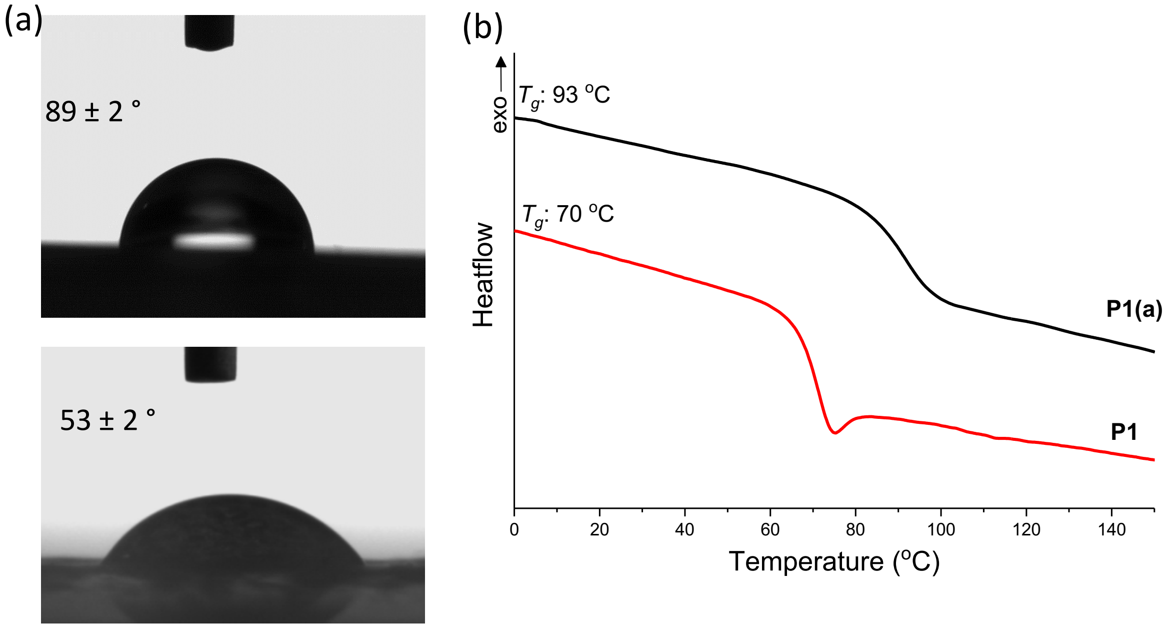

Figure S27. (a) Photographs of water droplets with their measured water contact angles of **P1** (top) and **P1(a)** (bottom). (b) DSC thermograms of **P1** and **P1(a)**, showing an increase in T_g after functionlization.

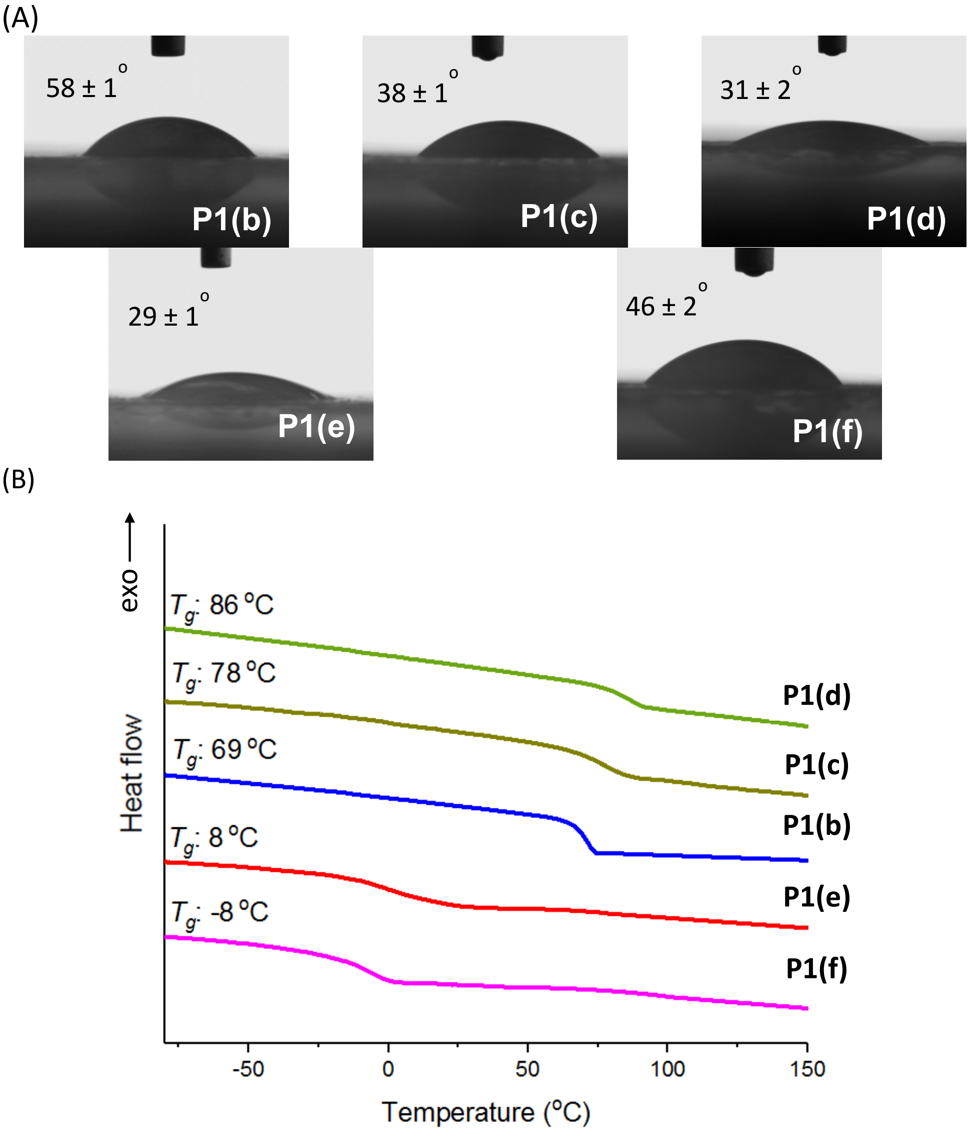

Figure S28. (A) Photographs of water droplets on polymer- coated glass surfaces (**P1(b)-P1(f)**). (B) Overlaid DSC traces of polymers **P1(b)-P1(f)**.

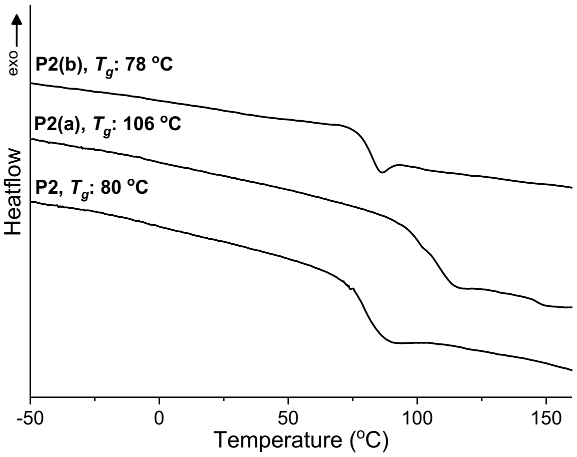

Figure S29. DSC thermograms of **P2**, **P2(a**) **and P2(b)**, showing the changes in T_g after functionalization.

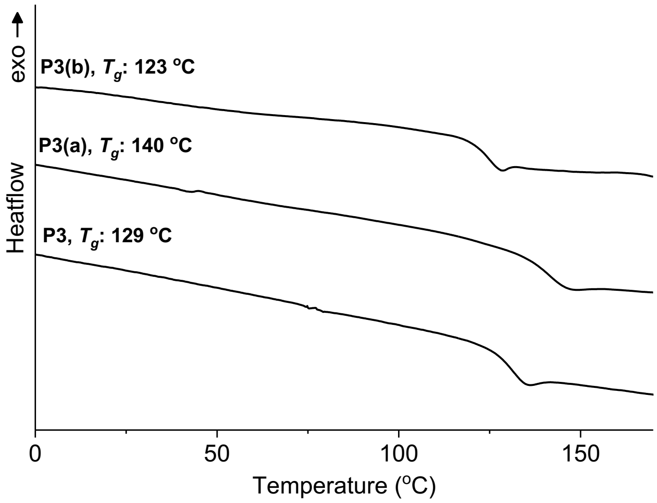

Figure S30. DSC thermograms of **P3**, **P3(a)** and **P3(b),** showing the changes in T_g after functionalization.

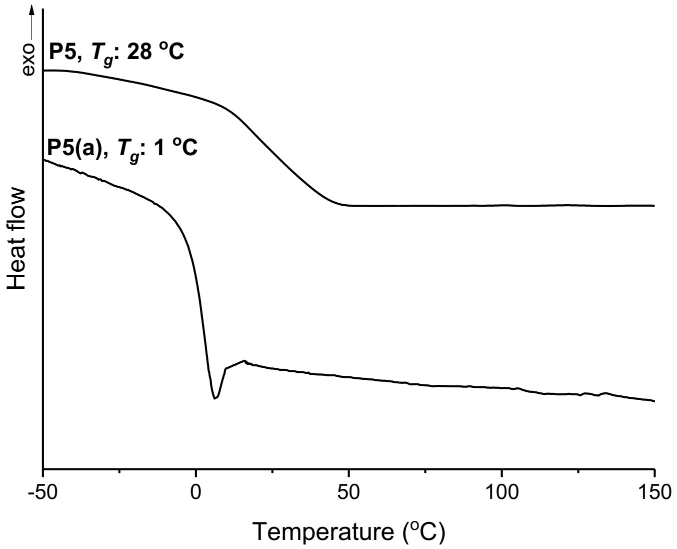

Figure S31. DSC thermograms of **P5** and **P5(a),** showing the change in T_g after functionalization.

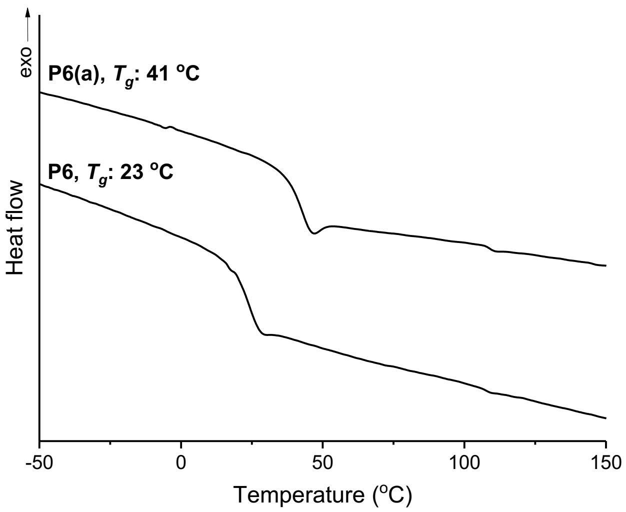

Figure S32. DSC thermograms of **P6** and **P6(a),** showing the change in T_g after functionalization.

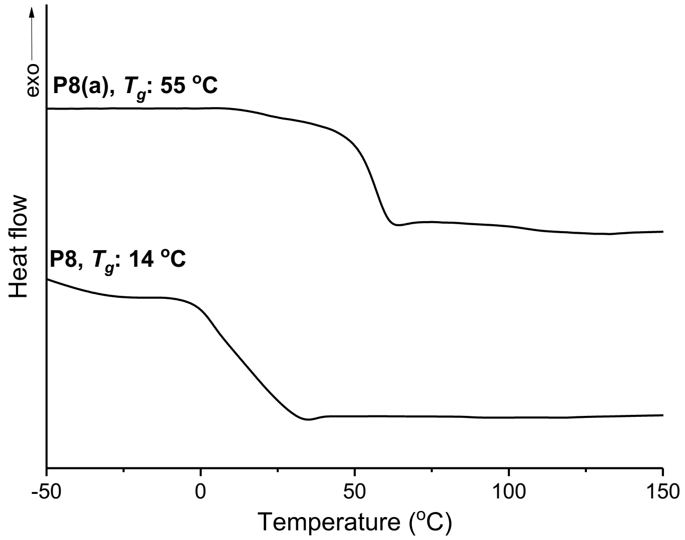

Figure S33. DSC thermograms of **P8** and **P8(a),** showing the change in T_g after functionalization.

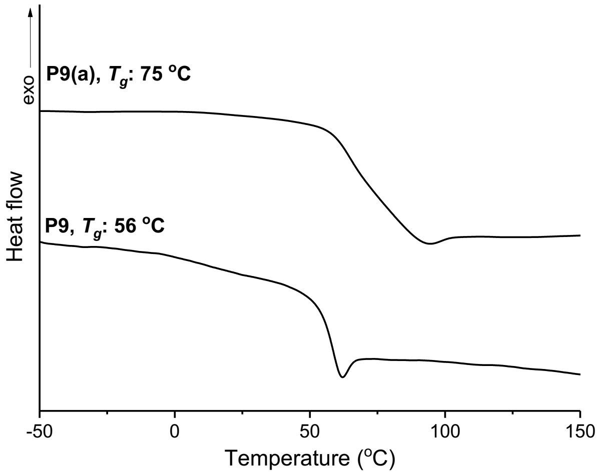

Figure S34. DSC thermograms of **P9** and **P9(a),** showing the change in T_g after functionalization.

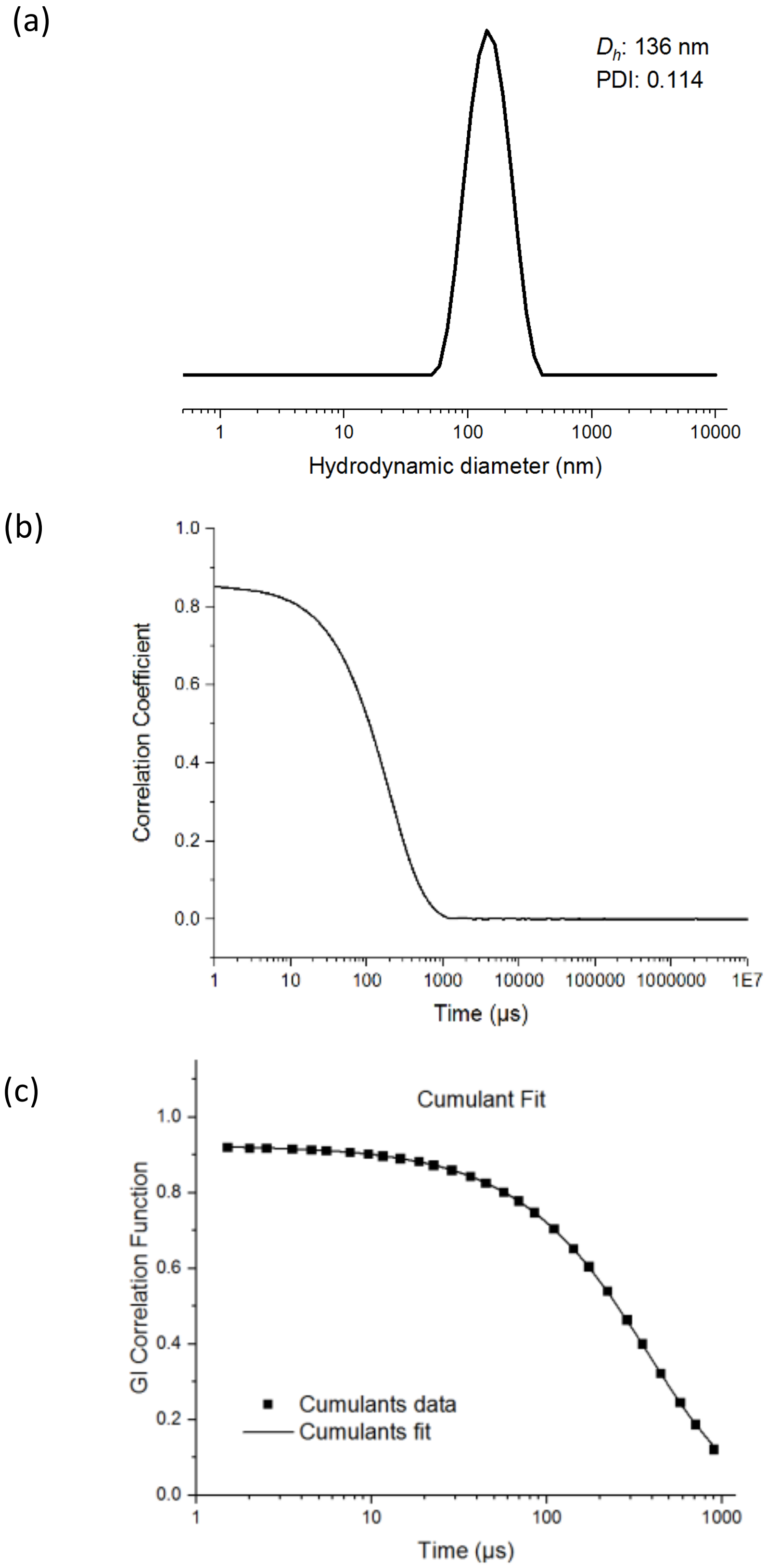

Figure S35. Dynamic light scattering data collected from an aqueous solution of **P1(b)** (10 $mgmL^{-1}$). (a) DLS data collected from an aqueous solution of 3 (5 $mgmL^{-1}$): Hydrodynamic diameter (D_h): $D_{h(intensity)}$= 136.0 ± 1.8 nm, and polydispersity index (PDI): PDI=0.11. (b) Cumulant fit and (c) Correlation functions of DLS study

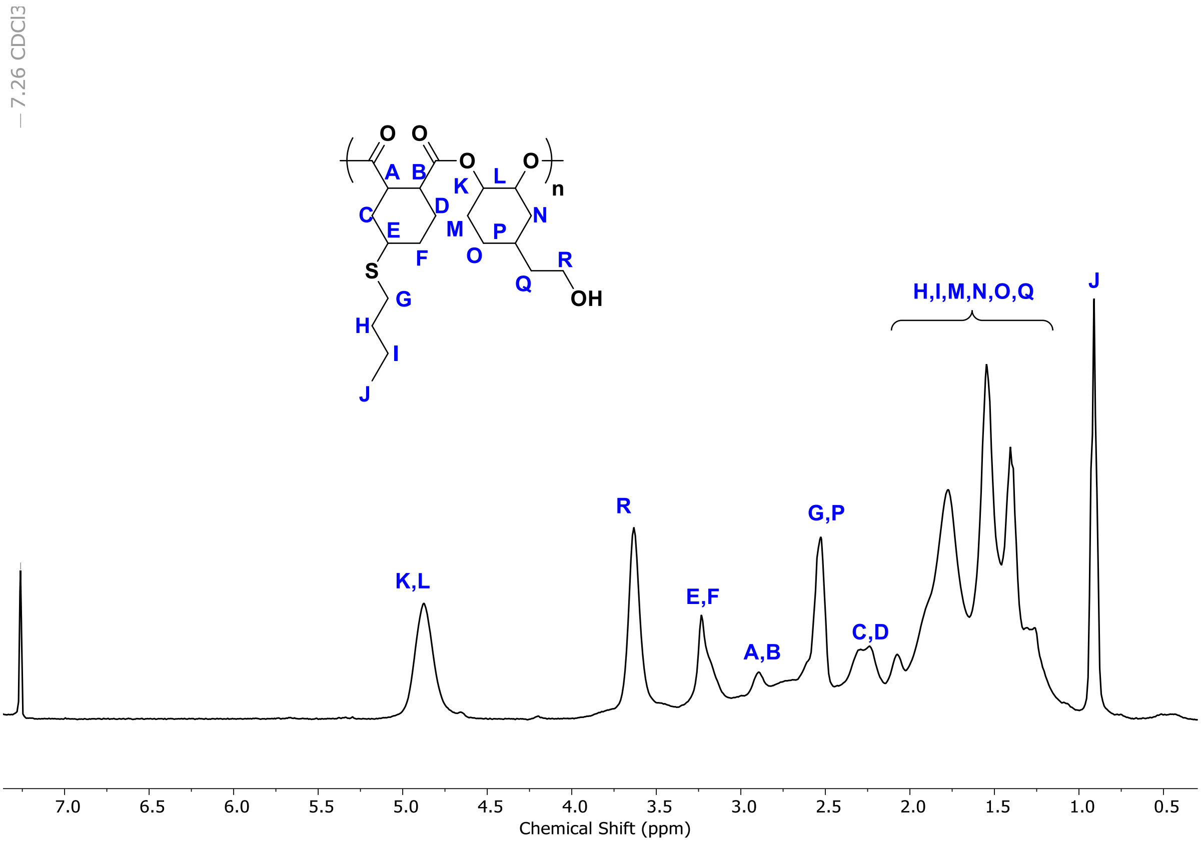

Figure S36. ^{1}H NMR spectrum of **P2(b)** ($CDCl_3$, 500.0 MHz, 298 K).

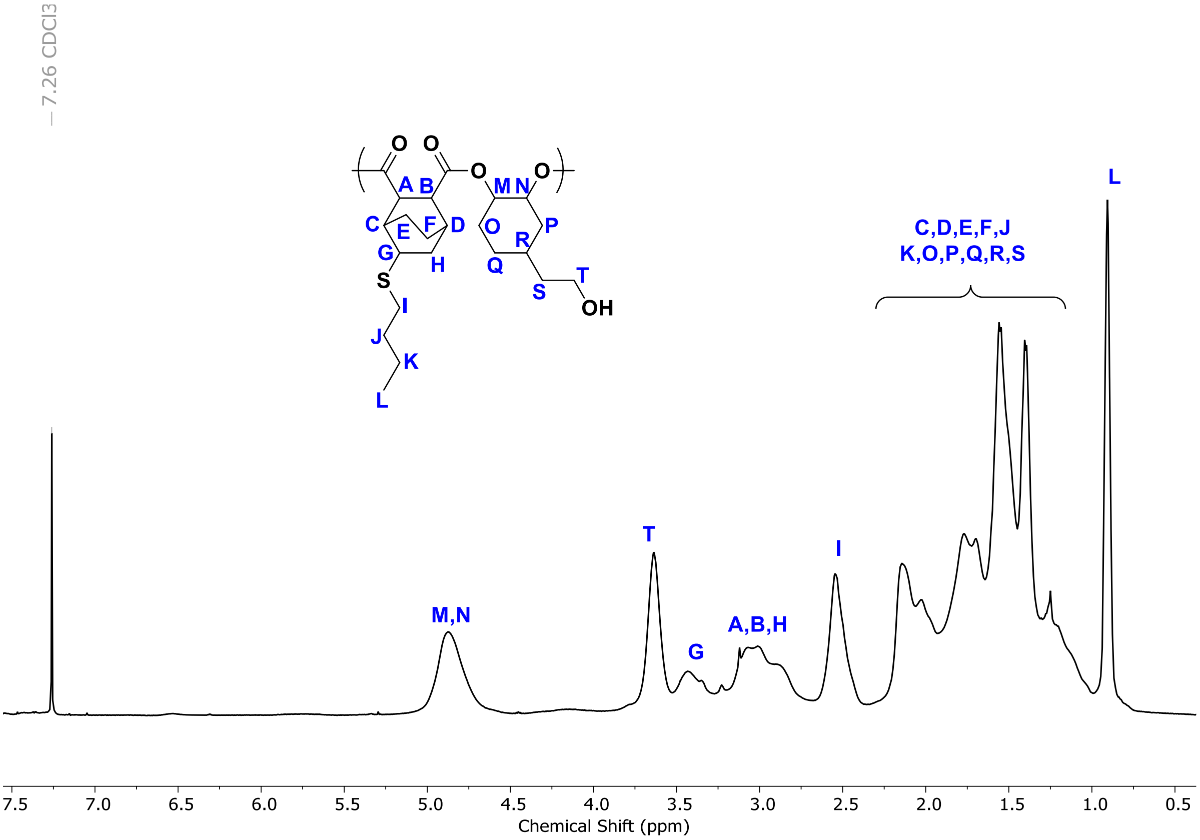

Figure S37. ^{1}H NMR spectrum of **P3(b)** ($CDCl_3$, 500.0 MHz, 298 K).

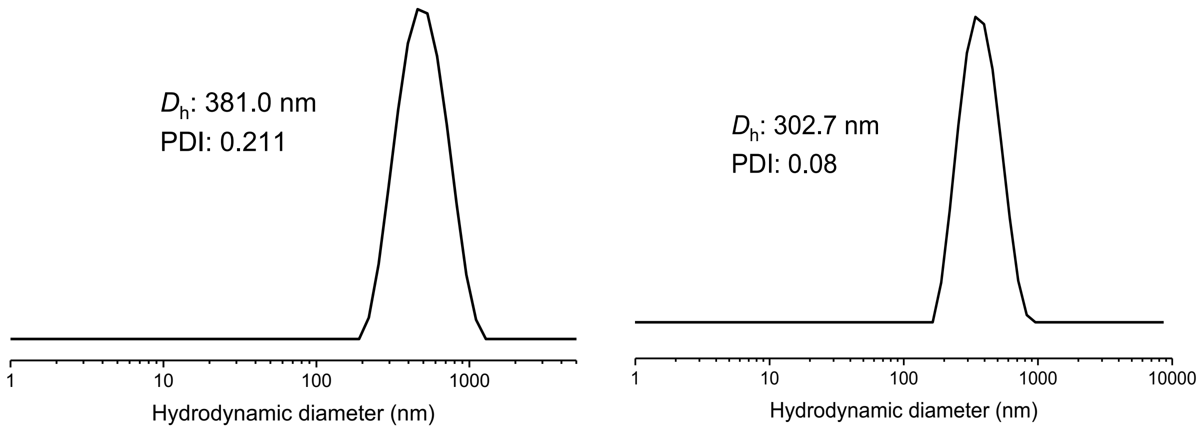

Figure S38. DLS data for self-assembly of **P2(b)** (left) and **P3(b)** (right).

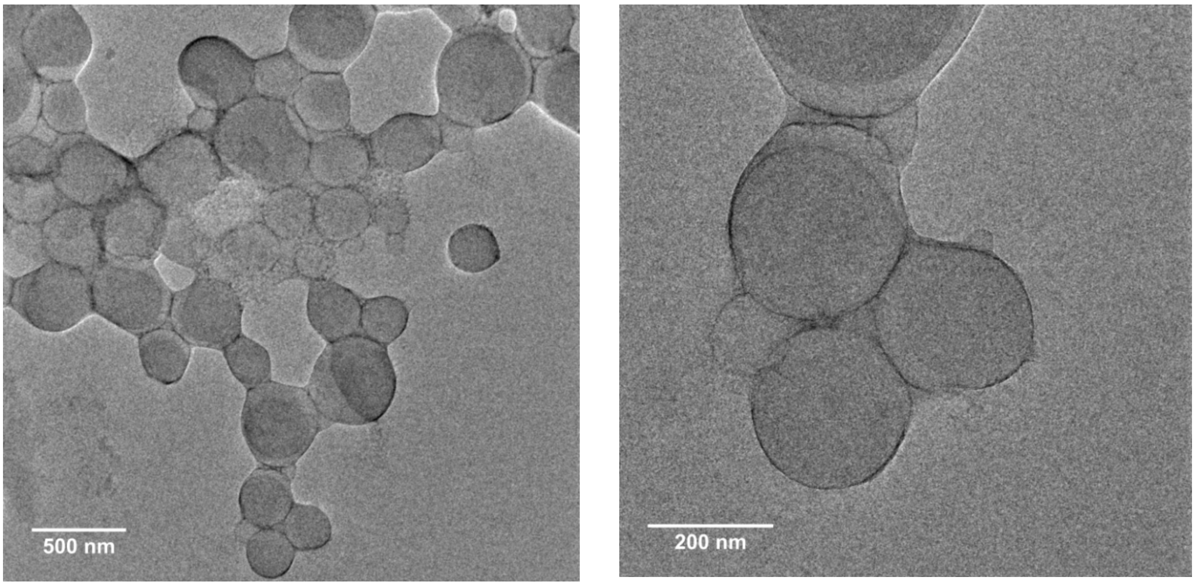

Figure S39. TEM images for the structure of the homopolymer micelle from **P2(b)**.

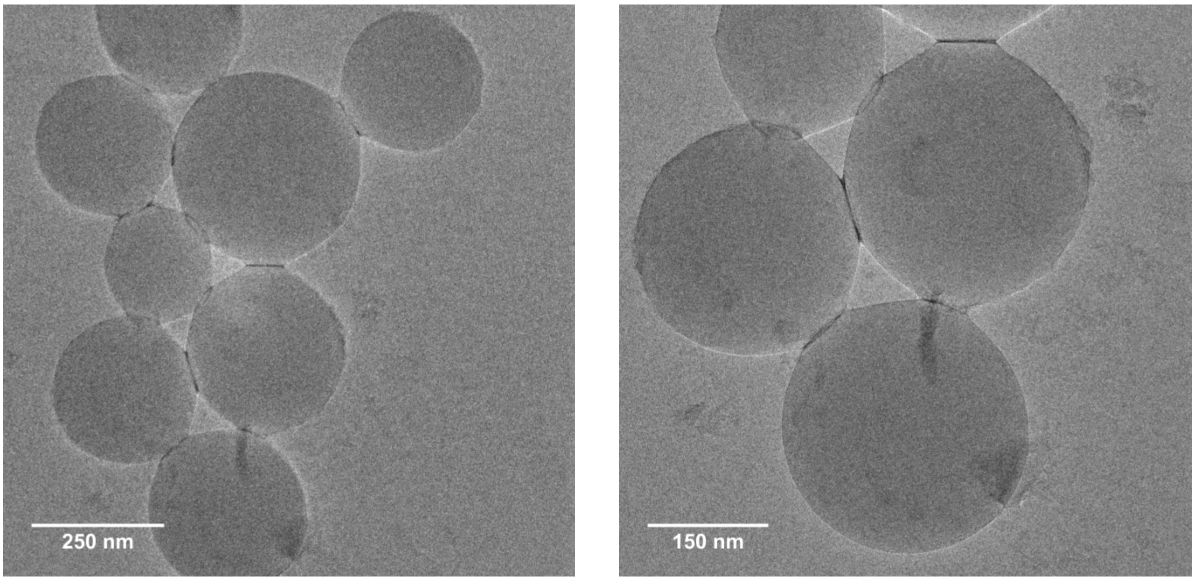

Figure S40. TEM images for the structure of the homopolymer micelle from **P3(b).**
